# Translocation effects on regional and local population viability and connectivity

**DOI:** 10.1111/cobi.70321

**Published:** 2026-05-12

**Authors:** Eva Sánchez Arribas, Aimara Planillo, Joe Premier, Tobias Kürschner, Elisa Belotti, Christine Breitenmoser‐Würsten, Luděk Bufka, Rok Černe, Thomas Engleder, Urša Fležar, Tomislav Gomerčić, Miha Krofel, Tereza Mináriková, Paolo Molinari, Simona Poláková, Kateřina Poledníková, Hubert Potočnik, Magda Sindičić, Ira Topličanec, Kristina Vogt, Josefa Volfová, Sybille Wölfl, Fridolin Zimmermann, Marco Heurich, Stephanie Kramer‐Schadt, Anja Molinari‐Jobin

**Affiliations:** ^1^ Department of Ecological Dynamics Leibniz Institute for Zoo and Wildlife Research (IZW) Berlin Germany; ^2^ Institute of Ecology Technische Universität Berlin Berlin Germany; ^3^ Department of Conservation Biology and Global Change Doñana Biological Station, (EBD‐CSIC) Seville Spain; ^4^ Department of National Park Monitoring and Animal Management Bavarian Forest National Park Grafenau Germany; ^5^ Department of Research and Nature Protection Šumava National Park Administration Kašperské Hory Czech Republic; ^6^ Faculty of Forestry and Wood Sciences Czech University of Life Sciences Prague Praha‐Suchdol Czech Republic; ^7^ Foundation KORA (Carnivore Ecology & Wildlife Management) Ittigen Switzerland; ^8^ Slovenia Forest Service Ljubljana Slovenia; ^9^ Lynx Project Austria Northwest Haslach an der Mühl Austria; ^10^ Biotechnical Faculty University of Ljubljana Ljubljana Slovenia; ^11^ Faculty of Veterinary Medicine University of Zagreb Zagreb Croatia; ^12^ ALKA Wildlife Dačice Czech Republic; ^13^ Faculty of Environmental Sciences Czech University of Life Sciences Prague Praha‐Suchdol Czech Republic; ^14^ Italian Lynx Project Tarvisio Italy; ^15^ Biology Centre CAS, Institute of Entomology České Budějovice Czech Republic; ^16^ Friends of the Earth Czech Republic, Carnivore Conservation Programme Olomouc Czech Republic; ^17^ WildLink Institute Waldmünchen Germany; ^18^ Department of Ecology and Evolution, Biophore, Quartier Sorge University of Lausanne Lausanne Switzerland; ^19^ Interdisciplinary Centre for Mountain Research University of Lausanne Lausanne Switzerland; ^20^ Chair of Wildlife Ecology and Wildlife Management, Faculty of Environment and Natural Resources University of Freiburg Freiburg Germany; ^21^ Institute of Forestry and Wildlife Management University of Inland Norway Koppang Norway

**Keywords:** Europe, large carnivore conservation, *Lynx lynx*, metapopulation, population viability, spatially explicit individual‐based model, translocations, Conservación de carnívoros mayores, Europa, *Lynx lynx*, metapoblación, modelo espacialmente explícito basado en individuos, translocaciones, viabilidad poblacional

## Abstract

Translocations and reintroductions aim to improve the viability of isolated populations and promote connectivity for large carnivores. However, there is no established framework for assessing their success. We used the Eurasian lynx (*Lynx lynx*) in western and central Europe to assess the impact of translocations on the viability of six populations and their interconnectivity. Lynx populations are small and isolated and have low genetic diversity. Population reinforcements have been carried out, but their impact has not been determined. We devised a workflow to evaluate how releasing new individuals affects population extinction probability and connectivity and patch colonization, and to estimate the minimum release population to create a stepping‐stone network. To this end, we calibrated a spatially explicit, individual‐based model with space use, survival, and reproductive rates observed in the populations and validated it with field data on population growth and expansion from the 1970s to 2020. We ran the model with the observed population structure in 1995−1996 to 2040 in two scenarios, with and without translocations. In both scenarios, most existing populations remained stable in our model for the next 20 years, but had low connectivity. Lynx translocations positively affected viability and connectivity on a population scale but did not increase connectivity between the eastern and western Alps. To create a stepping‐stone population that would significantly improve connectivity across the Alps, we conservatively estimated that at least 13 males and 37 females should be released in the Italian southeastern Alps based on the assumption of negligible immigration rates between patches. Concerted transboundary management to improve the future survival of lynx populations in western and central Europe is still needed.

## Abstract (es)

Efectos de la translocación sobre la viabilidad y la conectividad de las poblaciones regionales y locales

**Resumen:**

Las translocaciones y las reintroducciones tienen como objetivo mejorar la viabilidad de las poblaciones aisladas y promover la conectividad de los grandes carnívoros. Sin embargo, no existe un marco establecido para evaluar su éxito. Utilizamos el lince euroasiático (*Lynx lynx*) en Europa occidental y central para evaluar el impacto de las translocaciones en la viabilidad de seis poblaciones y su interconectividad. Las poblaciones de lince son pequeñas y aisladas, y presentan una baja diversidad genética. Se han llevado a cabo refuerzos de población, pero no se ha determinado su impacto. Diseñamos un flujo de trabajo para evaluar cómo la liberación de nuevos individuos afecta a la probabilidad de extinción de la población, a la conectividad y a la colonización de fragmentos, y para estimar la población mínima de liberación necesaria para crear una red de corredores discontinuos. Para ello, calibramos un modelo basado en individuos y espacialmente explícito con las tasas de uso del espacio, supervivencia y reproducción observadas en las poblaciones, y lo validamos con datos de campo sobre el crecimiento y la expansión de las poblaciones entre 197x y 2020. Aplicamos el modelo con la estructura poblacional observada entre 1995‐1996 y 2040 en dos escenarios: con y sin translocaciones. En ambos escenarios, la mayoría de las poblaciones existentes se mantuvieron estables en nuestro modelo durante los siguientes 20 años, pero presentaban una baja conectividad. Las translocaciones de linces afectaron positivamente a la viabilidad y la conectividad a escala poblacional, pero no aumentaron la conectividad entre los Alpes orientales y occidentales. Para crear una población puente que mejorara significativamente la conectividad a través de los Alpes, estimamos de forma conservadora que deberían liberarse al menos 13 machos y 37 hembras en los Alpes del sureste de Italia, partiendo de la hipótesis de que las tasas de inmigración entre fragmentos son insignificantes. Sigue siendo necesaria una gestión transfronteriza concertada para mejorar la supervivencia futura de las poblaciones de lince en Europa occidental y central.

## INTRODUCTION

Animal reintroductions and translocations for population reinforcements (i.e., the release of animals into an area where the species has been extirpated or the release of individuals from one area into an existing population to increase population viability, respectively [IUCN/SSC, 2013]) are increasing globally, although they are costly (Berger‐Tal et al., 2020; IUCN/SSC, 2013). Their main goals are restoring ecosystem processes, improving the conservation status of endangered species (Bubac et al., 2019), and promoting population connectivity (Peters et al., 2015). The latter is pivotal for animal populations inhabiting fragmented landscapes, especially where anthropogenic and natural barriers impede natural connectivity, as is the case for large carnivores in Europe (Zimmermann et al., 2007). Even after the recovery of some large carnivore populations (Cimatti et al., 2021), many have not achieved Favorable Conservation Status (i.e., a viable, self‐sustaining population in its natural habitat [European Commission, 1992]), and populations remain small and isolated (Chapron et al., 2014). If populations remain isolated for too long, demographic stochasticity and loss of genetic diversity due to inbreeding and genetic drift (Frankham, 2010) can lead to population decline (Pérez‐Pereira et al., 2022). Thus, conservation efforts focus on increasing population connectivity, and managers require tools to understand population responses and assess the probability of success of costly actions.

The key to maintaining viable wild populations in fragmented landscapes is to establish a metapopulation structure (García‐Antón et al., 2021). Metapopulations connected via natural emigration and recolonization processes can have an increased effective population size and reduced local extinction rate, increasing their viability (Hanski, 1998). The creation of one or more stepping‐stone populations can connect populations (Molinari et al., 2021; Saura et al., 2014). Especially for Eurasian lynx (*Lynx lynx*) (hereafter *lynx*), stepping‐stone populations are needed to ensure their long‐term viability in the highly fragmented European landscape. The a priori assessment of the likelihood of success of reintroductions and population reinforcements is mandatory for the long‐term survival of the species and must be followed by post‐release monitoring to evaluate success (Berger‐Tal et al., 2020).

Current western and central European established lynx populations emerged from multiple reintroductions in the 1970s, 1980s, and 2000s (see below) after the species went locally extinct (Boitani & Linnell, 2015). In the first two decades after the reintroductions, lynx started to naturally recolonize their former range (e.g., lynx from the Dinaric population sporadically reached the southeastern Alps in Slovenia, Austria, and Italy) (Čop & Frković, 1998; Molinari, 1998). Yet, effective population connectivity was never established, and population growth halted in the 1990s in most lynx populations (von Arx et al., 2021). Twenty years after the last lynx were released, all reintroduced populations are still small and far from long‐term viability. In most western and central European populations, viability is jeopardized by human‐induced mortality (e.g., vehicle collisions, illegal hunting) (Heurich et al., 2018; Vogt et al., 2025) and low genetic diversity (Gajdárová et al., 2023; Mueller et al., 2022). Inbreeding levels were high before recent reinforcement programs in the Dinaric (Skrbinšek et al., 2019), Jura, and northwestern alpine populations (Mueller et al., 2022).

In conservation, a common approach is the 50−500 rule of thumb, stating that 50 reproducing individuals are needed to avoid extinction due to inbreeding depression in the short term. An effective population size of 500 individuals is required for long‐term evolutionary potential (Franklin, 1980). But in high human density areas like western and central Europe, finding large enough habitat patches to accommodate the 50−500 rule of thumb is very challenging, and this goal can only be achieved by a functional metapopulation (Molinari et al., 2021). For this purpose, additional lynx reintroduction and reinforcement projects took place in the past two decades, in Austria (Fuxjäger, 2012), Germany (Idelberger et al., 2021; Nationalpark Harz, 2023), and Switzerland (Foundation KORA, 2023, 2024), among others.

In the southeastern Alpine and Dinaric lynx populations, two reinforcement projects started in 2013 (ULyCA, Urgent Lynx Conservation Action in Italy) and 2017 (LIFE Lynx Project, LIFE16 NAT/SI/000634 in Slovenia, Italy, and Croatia) to improve their genetic status and create a stepping‐stone population in the southeastern Alps. From 2019 to 2023, 23 lynx were translocated to the Italian southeastern Alps (*n* = 5), the Slovenian southeastern Alps (*n* = 6), and the Dinaric population in Slovenia and Croatia (*n* = 12) to reinforce the Dinaric and southeastern Alpine population. But observing the demographic effects of these projects on the population might take many years.

We used a modeling tool to evaluate the impact of lynx translocations in the Dinaric Mountains and southeastern Alps (SE Alps) on the viability and connectivity of lynx populations. We also estimated the characteristics of a stepping‐stone system that would increase connectivity between lynx populations in western and central Europe. We included the Jura Mountains, the northwestern Alps (Switzerland), the Bohemian‐Bavarian‐Austrian Forest (BBA), and the Austrian Alps. We excluded the recently established lynx populations in the Palatinate Forest and northern Vosges (Idelberger et al., 2021) because we focused on the Alps and neighboring populations.

We devised a framework to assess management success by integrating monitoring data from the 1970s until 2020 (model calibration and scenarios’ section) at large spatial scales in a spatially explicit individual‐based model tailored to lynx (SE‐IBM) (Kramer‐Schadt et al., 2004, 2005, 2007). We evaluated the predicted success of conservation interventions based on the differences between a future projection without conservation intervention as a baseline (scenario 1) and a scenario that considered population reinforcement through lynx translocations conducted from 2019 to 2023 (scenario 2), reflecting the ULyCA2 and LIFE Lynx conservation interventions. Our goal was to predict the impact of the recent population reinforcement on population development and connectivity, and assess further management needs to create a large spatially structured lynx population system in western and central Europe. We expected a positive effect of the population reinforcements on population size in the southeastern Alps and Dinaric lynx populations and thus a decrease in extinction risk, as well as an increase in connectivity from the southeastern Alps to the Dinarics and surrounding areas like the central eastern Alps in Italy and the eastern Alps in Austria. We sought to provide a blueprint of how to use spatially explicit predictive models for species conservation and to support adaptive management of populations. Furthermore, our framework can be adapted to other species by modifying population parameters, thus serving as a tool for conservation relevant for many ecological systems.

## METHODS

### Model overview

The essence of the SE‐IBM is to track each individual throughout its lifetime in the landscape. To model all individual‐level processes of survival, reproduction, dispersal, and territory settlement, the original lynx SE‐IBM (Kramer‐Schadt et al., 2004, 2005) comprised a demographic and a dispersal submodel (Appendix S1). The demographic submodel accounted for life‐history processes (birth, mortality, and reproduction) and territory establishment. For this study, we calibrated the demographic submodel with lynx data from field studies in the study area in western and central Europe (Appendices S2 & S3). The dispersal submodel simulated individual movement across the landscape, based on radio telemetry data from Switzerland from 1988 to 1991 (Breitenmoser et al., 1993).

**FIGURE 1:**
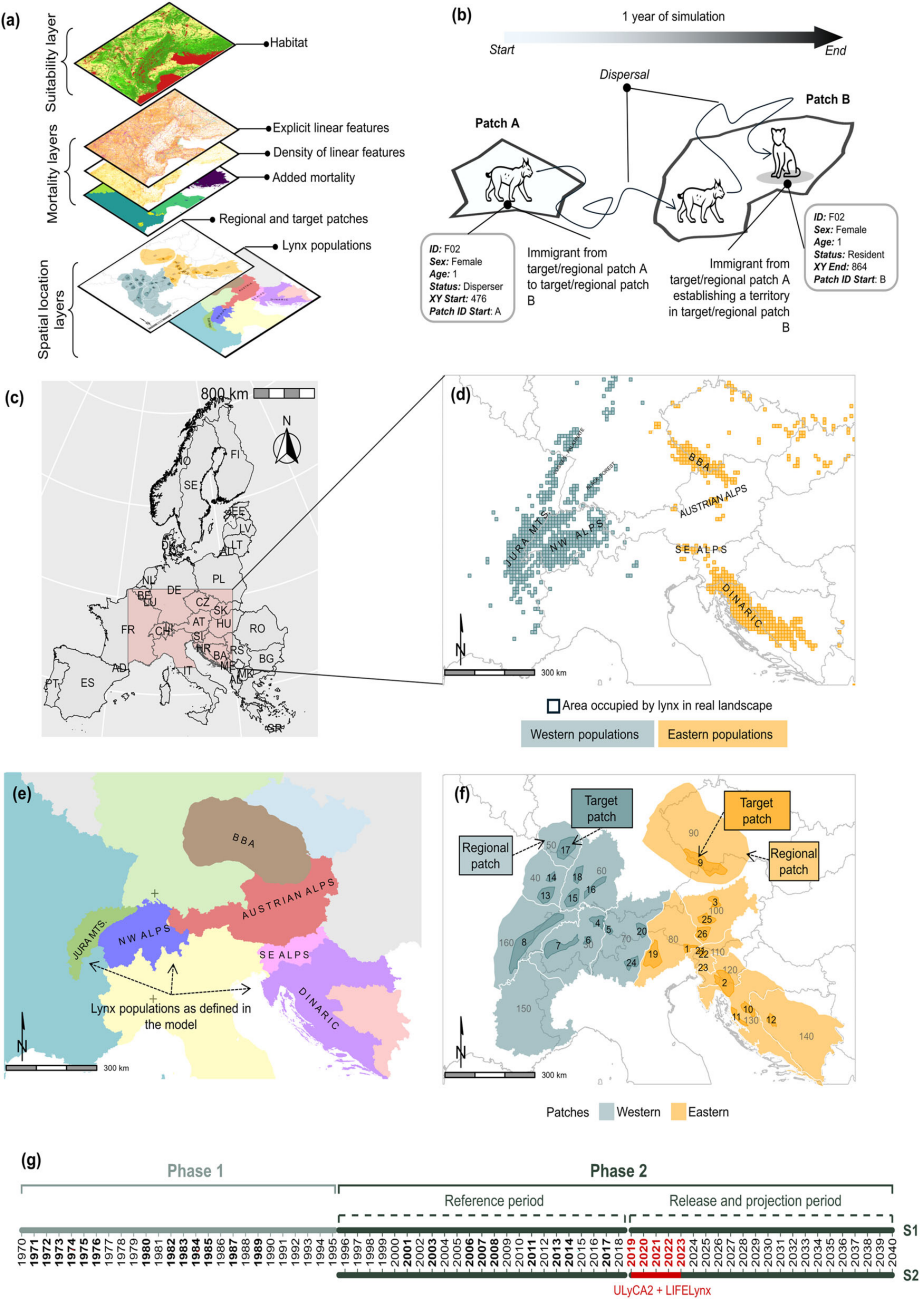
(a) Layers used in the spatially explicit individual‐based model of six lynx populations in central Europe that tracks lynx throughout their lifetime (reference layer, suitability layer; mortality layers, mortality probability of the modeled lynx based on multiple sources; spatial location layers, auxiliary layers used to track individuals in space); (b) movement of an individual lynx from patch A (target or regional born in that patch) to patch B (target or regional), where it becomes a resident within a year of simulation; (c) study area (red) in Europe in the simulation; (d) distribution of lynx populations modified from Kaczensky et al. (2021) (squares, area occupied by lynx); (e) lynx populations as defined in the model; (f) spatial location layers showing the nested distribution of target patches (1−26 darker color, areas of high lynx density and areas of importance for connectivity) within regional patches (30−160 lighter color, large areas surrounding target patches); and (g) visual representation of timeline from the first lynx reintroductions (years in bold) in the 1970s to the end of the simulation (timeline shows distribution of phases 1 and 2, "reference period" and "release and projection period" in phase 2, and scenarios S1 [absence of ULyCA2 and LIFE Lynx population reinforcements] and S2 [ULyCA2 and LIFE Lynx population reinforcements, red color]).

The model ran on spatial raster layers, each with a 1‐km^2^ cell resolution, grouped into three categories: a habitat suitability layer, mortality layers, and spatial location layers (Figure 1a). The habitat suitability layer dictated the dispersal process and territory settlement process of individuals in the landscape (Schadt et al., 2002). The mortality layers influenced the survival probability of dispersing and resident individuals. The spatial location layers classified the landscape into patches, useful to define the spatial extent of lynx populations in the model or to delineate specific areas in the landscape important for lynx connectivity, for example. The spatial location layers were thus used to count the lynx recorded in a specific area and analyze the dynamics in that area. Although the habitat and mortality layers affect demographic and dispersal processes, the spatial location layers were only used to locate individuals in the landscape (Figure 1a). Spatial location layers did not influence the movement of individuals, meaning individuals that travel through the landscape may have entered or left patches without an effect on their movement or survival. For detailed information on each of the spatial layers, see Appendix S4.

For every simulated year, the model recorded intrinsic parameters associated with every individual, including its individual identity (ID), age, sex, status (resident or disperser), ID of the mother and the father, territory size (for residents only), fate (alive or dead), and cause of mortality (baseline, traffic, or additional mortality). The model recorded the coordinates where the individual was born, the coordinates where the individual was at the beginning and end of each year, and the coordinates where the animal died, if applicable. For each coordinate recorded, the model also recorded the area associated with these cells as delineated by the spatial location layers, for example, in which population the lynx could be found (Figure 1e) and which regional or target patch number (patch ID) the lynx location belonged to (Figure 1f). Therefore, by recording all the individual‐level processes that take place in the model, we derived population‐level metrics like demographic development and connectivity between patches.

### Study area and spatial scales

Our study area extended across western and central Europe, from France to the west to Croatia on the east. It included the current lynx populations in the Jura Mountains of Switzerland and France, the northwestern Alps (Switzerland), the BBA, Austria (Kalkalpen), the southeastern Alps (including Italy, Slovenia, and Austria), and the Dinaric Mountains of Slovenia and Croatia (Figure 1c,d). Forest covered up to 80%, and human population density varied from 2 to 140 inhabitants/km^2^ (Appendix S5). Based on historic population patterns and lynx reintroduction history, we classified lynx populations into west and east for visualization purposes (Figure 1d).

In the model, we included two different spatial location layers for our study area. The first spatial location layer was referred to as the lynx population layer (Figure 1e) and delineated the boundaries of the current lynx populations as described in the literature (Mináriková et al., 2023; Pazhenkova et al., 2025; Zimmermann & Breitenmoser, 2007; Zimmermann et al., 2011) and observed population distribution (Figure 1d) (Kaczensky et al., 2021). This layer represented the extent of the real lynx populations in our model. The second spatial location layer divided the landscape into patches with a nested design of smaller patches defined within larger patches (Figure 1f). This layer was referred to as the patch connectivity layer. The small patches (hereafter *target patches*) consisted of 26 patches defined according to areas important for lynx connectivity (Figure 1f) from around 113−4608 km^2^ distributed across the landscape. Target patches 3, 8, 7, 9, and 2 represented the core areas (areas in the population with the highest lynx density) of the Austrian, Jura, northwestern Alps, BBA, and Dinaric lynx populations, respectively. The other target patches represented potential sites for creating stepping‐stone populations according to expert knowledge of the species. The larger patches (hereafter *regional patches*) represented larger areas (8440–64,178 km^2^) surrounding the target patches, dividing the landscape into coarse geographic regions. For example, regional patches 150 and 30 represented the western Alps, the central western Alps were essentially defined as regional patch 70, regional patch 80 represented the central eastern Alps, and regional patch 100 represented the eastern Alps. Simultaneously, regional patch 70 (central western Alps) included the target patches 5, 20, and 24 (Figure 1f). Therefore, target patches were used to capture specific dynamics inside regional patches. See Appendix S6 for the relation between the lynx population layer, patch connectivity layer, and the geographical region in the landscape.

### Model calibration and scenarios

We calibrated our model by modifying the demographic‐related parameters (Appendix S2) to match the published lynx population sizes and expansion patterns since the reintroductions and subsequent reinforcements until 2020 (Appendices S9 & S10 & S11). For the calibration process, we used the lynx population spatial location layer in the model (Figure 1e). For each of our lynx population patches, we extracted the average (SD) and median number of residents, dispersers, and total population size (lynx and dispersers combined) and compared our output with the reported population sizes from field reports and publications (Appendix S9). We also compared the population expansion of each lynx population in our model with the real distribution of the respective lynx populations to validate the model (Appendix S10).

To replicate the phases of expansion following reintroductions, and the later period of stagnation of lynx populations, we distinguished two phases during the model calibration. These phases were differentiated by the change in the mortality‐related demographic parameters of the model (CorrFactDisp, CorrFactRes, and Added_mort) (Appendix S2, Heurich et al., 2018), which led to changes in survival and, therefore, changes in the demographic parameters of our model. Because the demographic parameters CorrFactDisp and CorrFactRes remained constant throughout the simulation, phases 1 and 2 were modeled separately to adapt these parameters and calibrate the model accordingly.

In our model, phase 1 (1970–1995/96) was related to the reintroductions and initial population expansion of lynx populations, and it was thus characterized by high survival and high population growth rates leading to a range expansion of lynx (Appendix S2). Only the successful reintroductions and population reinforcements in the 1970s and 1980s were used as the starting point of the simulated populations in phase 1 until the start of the stagnation phase (Appendices S7 & S8). We calibrated the parameter values until our simulations fitted the observed population sizes and expansion patterns at the end of phase 1.

Then, we used the resident spatial distribution of lynx territories at the end of phase 1 as the initial population distribution and size (number of residents = number of territories) for phase 2 with a 3‐year burn‐in period. Therefore, the output of phase 1 was used as the input for phase 2 to maximize the continuity of the development of lynx populations in our model. We also included the lynx reintroductions and population reinforcements that took place in the different lynx populations related to phase 2 (Appendices S7 & S8). Phase 2 (1996/97–2040) represented population growth halt and future projection and thus had lower survival and lower growth rates and a halt of range expansion in most populations (Breitenmoser et al., 1998; Fležar et al., 2021; Heurich et al., 2018) (Appendix S3). We distinguished and analyzed two periods in phase 2: reference period (1996–2018), used to validate the model against observed lynx data, and a release and population projection period (2019−2040), used to predict lynx population developments (Figure 1g). The population projection period is a direct continuation of the reference period, and both were included in the same simulation run with the same demographic parameters. For the predictions in this second projection period of phase 2, we used two contrasting scenarios. Scenario 1 was without reinforcement measures and did not account for the 23 lynx translocations effectuated from 2019 to 2023 in the Dinaric and southeastern Alps by ULyCA2 and LIFE Lynx projects, whereas scenario 2 included them. The difference between scenario 1 and scenario 2 relied only on the absence or presence of reintroductions, whereas the demographic parameters were the same in both scenarios; hence, the differences between the two scenarios could only be related to the reintroductions. We ran each simulation 100 times to account for variability and stochasticity in the model and averaged the results. We focused on phase 2 of the simulations, especially on the release and population projection period.

### Population viability analyses

For each population and year of phase 2, we calculated the extinction probability (*p*_ext_), population size, and population growth parameters according to the lynx population layer (Figure 1e). Extinction probability was calculated as the proportion of repetitions where the population size was below the quasiextinction threshold of 50 resident lynx, reflecting the rule of thumb (Franklin, 1980) (i.e., a population was considered extinct if the number of resident lynx was <50). Population size was provided by the average number of simulated resident individuals in a given population each year. Growth parameters were the average population growth rate (μ) and the growth rate variation (σ^2^), calculated with a 95% confidence interval following the framework of Morris and Doak (2002). A population tends to decline if μ < 0, to be stable if μ = 0, and to grow if μ > 0 (Morris & Doak, 2002). All parameters were calculated for the reference period (1995–2018) and the release and projection periods (2019−2040) for scenarios 1 and 2.

### Connectivity analyses

Connectivity was measured according to the patch connectivity layer at the two patch scales: target patch scale and regional patch scale (Figure 1f). For each scale, we measured connectivity with two metrics. We measured the patch connectivity rate (*p*_conn_) in scenarios 1 and 2 by calculating the average rate of annual immigration between patches at the corresponding scales (Figure 1f) for both periods and scenarios. We defined the connectivity rate between two patches at a year *t* (*p*_conn_ *_y_*_,_ *_x_*) as the proportion of repeated simulation runs with immigration success at year *t*. We defined immigration success at patch *y* as the arrival of at least one lynx coming from patch *x* and not born in patch *y*. Thus, connectivity between two patches for any given period was the average of the connectivity (*p*_conn_ *_y_*_,_ *_x_*) for that period.

Connectivity was also measured as the immigration effect on the number of resident lynx and thus territory establishment (scenario 2) in the 26 target patches from the patch connectivity layer (Figure 1f), because we expected an overall positive effect of immigration on the number of residents. This would indicate that immigrant lynx arriving at a target patch would establish a territory. To understand how immigration affects the number of residents in target patches, we tested the influence of the number of immigrants arriving at a patch at year *t* (immigration load) on the population size of residents at *t+*1. We expected patch population size to be temporally correlated due to demographic processes taking place (offspring from resident individuals establishing a territory in the same patch when available) and immigration processes due to dispersal (immigrant lynx establishing a territory in a patch when available) taking place. Therefore, we fitted a linear mixed‐effects model with temporal autocorrelation and a Gaussian error distribution. Our dependent variable was the population size of residents at *t*+1. As fixed effects, we included year, patch size to account for available habitat for lynx to establish a territory, number of immigrants (total immigration load) at *t*, and its interaction with patch size. We included target patch ID as a random intercept to account for additional factors that may influence the relationship between immigration and population size of residents, such as the location of the patch. For example, target patches in the core areas (patch IDs 2, 3, 7, 8, and 9) of the real lynx populations, and thus where most of the area is occupied by lynx, are likely to be less affected by immigration than patches in the periphery of populations that are only partially occupied (e.g., patch IDs 13, 15). We fitted the models with the function lme from the nlme package (Pinheiro et al., 2021) 3.1‐161 in R (R Development Core Team, 2022) and ran model diagnostics to ensure all assumptions were met.

### Minimum release population

After the translocations, we estimated the minimum number of female and male lynx to be released simultaneously in potentially suitable target patches from the patch connectivity layer, between eastern and western Alps, that will ensure population survival in any of the patches from 2023 until 2040 with at least 95% chance (*p*_ext_ < 0.05). We decreased the quasi‐extinction threshold for stepping‐stone populations to 20 lynx (Thor & Pegel, 1992) because the aim of these patches was to serve as refuge areas for lynx to promote migration to neighboring patches. A target patch was hence considered suitable as a stepping‐stone for releases when it had a carrying capacity (*K*) of ≥20 residents (details on territory settlement rules in Appendix S1; parameters in Appendix S3) and was located between at least two lynx populations (e.g., the northwestern Alps and Austrian lynx populations). Target patches ID 19, 20, 24, 25, and 26 (Figure 1f) fulfilled the requirements to be considered potential patches for reintroduction. To determine the minimum release population (MRP), we released an increasing number of females and males at each patch with a maximal sex ratio of 1:3, independently, until the number of females and males released equaled the carrying capacity of the patch, or until the simulated population reached *p*_ext_ < 0.05. We simulated all releases to occur in 2023. Releases in target patches were simulated independently, and the number of lynx at each target patch before the animals were released was zero or near zero. Therefore, the effect on extinction rates in our results is a consequence of the lynx releases.

## RESULTS

### Population viability

**FIGURE 2:**
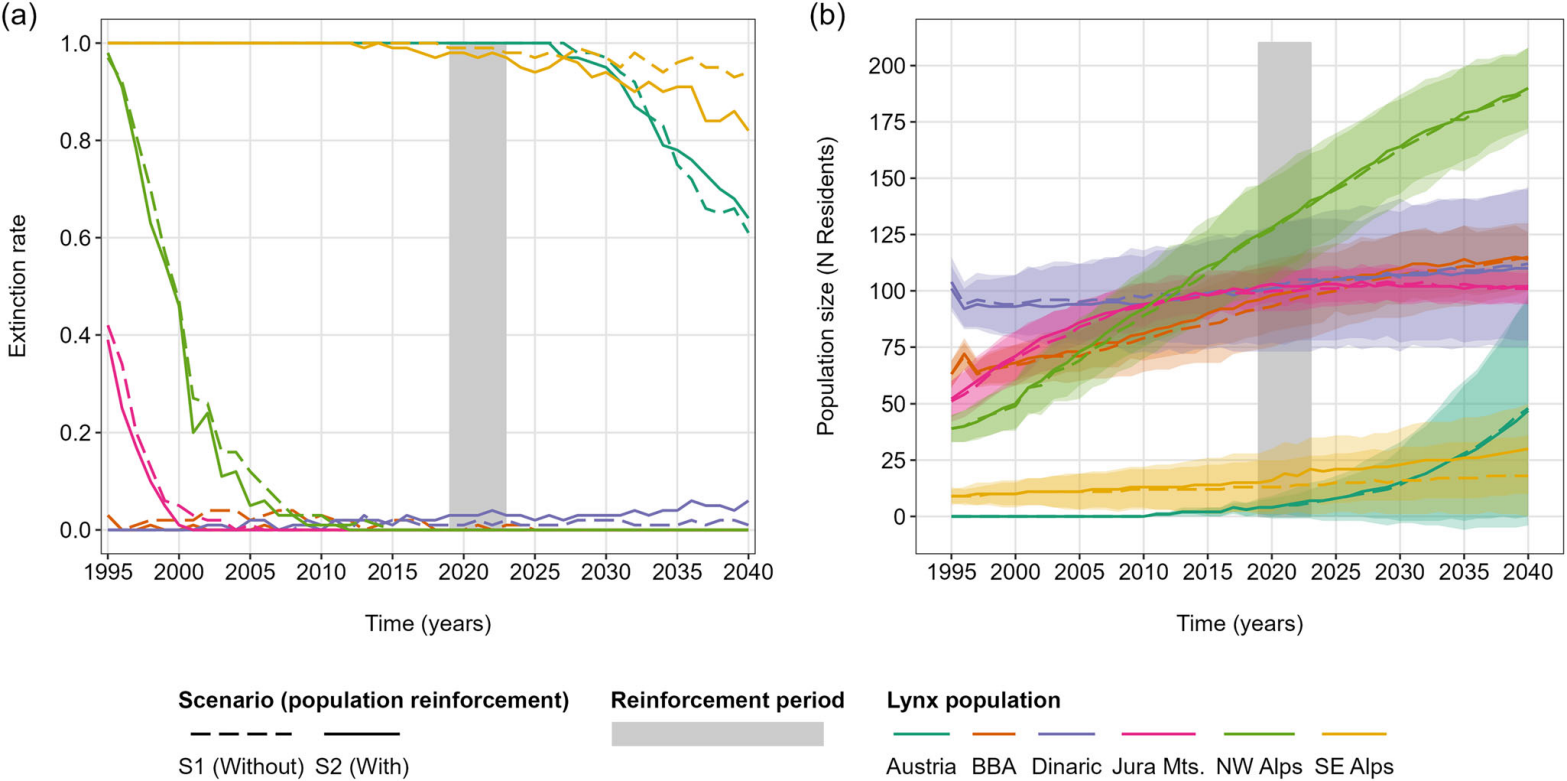
(a) Extinction rates and (b) mean population sizes of residents (SD) of different lynx populations (colors) simulated 100 times (dashed lines, simulation with absence of population reinforcements [scenario 1]; solid lines, simulation with lynx population reinforcements [scenario 2]; shaded area, period of population reinforcements).

Although extinction probability (*p*_ext_) in 2024 was <15% in the Jura, northwestern Alps, Dinaric, and BBA lynx populations, it was >50% in the Austrian and the southeastern alpine populations (Figure 2a). Lynx translocations had a notable positive effect in the southeastern Alpine population by decreasing the extinction risk; however, based on model forecasts, the lynx population did not reach 50 individuals by 2040 (Figure 2b). Without population reinforcement, the lynx would be functionally extinct in the southeastern Alps. The highest increase in population size was predicted for the Austrian population on average, although the SD overlapped zero, and the extinction rate was estimated at 60% (Table 1 & Figure 2b). In 2040, four populations were estimated to have >50 resident lynx, but only the northwestern Alpine population continued to grow. The BBA and Dinaric population showed a slight growth during 1995–2040, and the Jura population, although showing an increase at first, stabilized around the years 2015−2019 (Figure 2b).

**TABLE 1:** Diffusion parameters of sizes of resident lynx populations calculated for the reference period and the projected release period in a simulation for scenarios 1 (no ULyCA2 and LIFE Lynx^a^ translocations) and 2 (LIFE Lynx translocations).

Period	Population	Scenario	μ (CI)^b^	σ^2^ (CI)^c^
Reference (1995–2018)	Austria	1	0.0507 (−0.2814, 0.3828)	0.1578 (0.069, 0.6538)
	Bohemian‐Bavarian Austrian (BBA)	1	0.0151 (−0.0003, 0.0305)	0.0013 (0.0008, 0.0026)
	Dinaric	1	−0.0012 (−0.0123, 0.0098)	0.0007 (0.0004, 0.0013)
	Jura Mountains	1	0.0268 (0.0153, 0.0384)	0.0007 (0.0005, 0.0015)
	northwestern Alps	1	0.0468 (0.0322, 0.0614)	0.0012 (0.0007, 0.0023)
	southeastern Alps	1	0.0044 (−0.0278, 0.0366)	0.0058 (0.0035, 0.0114)
Projected release (2019–2040)	Austria	1	0.1154 (0.0709, 0.1599)	0.0101 (0.006, 0.0205)
	Austria	2	0.1076 (0.0119, 0.2033)	0.0466 (0.0276, 0.0951)
	Bohemian‐Bavarian Austrian (BBA)	1	0.0105 (0.0032, 0.0178)	0.0003 (0.0002, 0.0006)
	Bohemian‐Bavarian Austrian (BBA)	2	0.0068 (0.0001, 0.0135)	0.0002 (0.0001, 0.0005)
	Dinaric	1	0.0040 (−0.0055, 0.0134)	0.0005 (0.0003, 0.0009)
	Dinaric	2	0.0044 (−0.0065, 0.0154)	0.0006 (0.0004, 0.0012)
	Jura Mountains	1	0.0014 (−0.0034, 0.0061)	0.0001 (0.0001, 0.0002)
	Jura Mountains	2	−0.0004 (−0.0072, 0.0063)	0.0002 (0.0001, 0.0005)
	northwestern Alps	1	0.0202 (0.0151, 0.0254)	0.0001 (0.0001, 0.0003)
	northwestern Alps	2	0.0201 (0.0139, 0.0263)	0.0002 (0.0001, 0.0004)
	southeastern Alps	1	0.0083 (−0.0292, 0.0458)	0.0072 (0.0042, 0.0146)
	southeastern Alps	2	0.0473 (0.0064, 0.0883)	0.0085 (0.005, 0.0174)

^a^ The ULyCA2 and LIFE Lynx projects are reintroduction and translocation projects aiming to restore the southeastern and Dinaric lynx populations and enhance lynx connectivity in the eastern.

^b^ Average growth rate calculated with a confidence interval of 95%. Values of μ > 0 indicate population growth, μ < 0 indicates population decline, and μ = 0 indicates a stable population.

^c^ Growth rate variation. Increasing values of σ^2^ indicate an increasing uncertainty around μ.

Lynx translocations increased the average population growth rate (µ_scenario 1_ = 0.0083, µ_scenario 2_ = 0.0473, Table 1) and decreased the *p*_ext_ of the southeastern alpine lynx by ∼13%, from 0.94 in 2040 in scenario 1 to 0.82 in scenario 2 (Figure 2a). Translocations did not affect *p*_ext_ of the Dinaric population. The slight increase in *p*_ext_ in scenario 2, from 0.01 to 0.06 (Figure 2a), was not related to differences in growth rate between scenarios (µ_scenario 1_ = 0.0040; µ_scenario 2_ = 0.0044), although the rate of variation was higher in scenario 2 (σ^2^_scenario 1_ = 5e‐04; σ^2^_scenario 2_ = 6e‐04) (Table 1).

As expected, in the Jura, northwestern Alpine, Austrian, and BBA lynx populations, *p*_ext_ and population size varied little between scenarios (Figure 2), all with positive or stable population growth parameters (Table 1). However, in the release and projection period (2019–2040), the average population growth was lower and the rate of variation higher than in the reference period (1995/96–2018) (Table 1). In the Jura population, the average growth rate stabilized in the projection period, whereas the population increased by 2%−3% in the reference period, until 2019 (Table 1).

### Connectivity analyses

**FIGURE 3:**
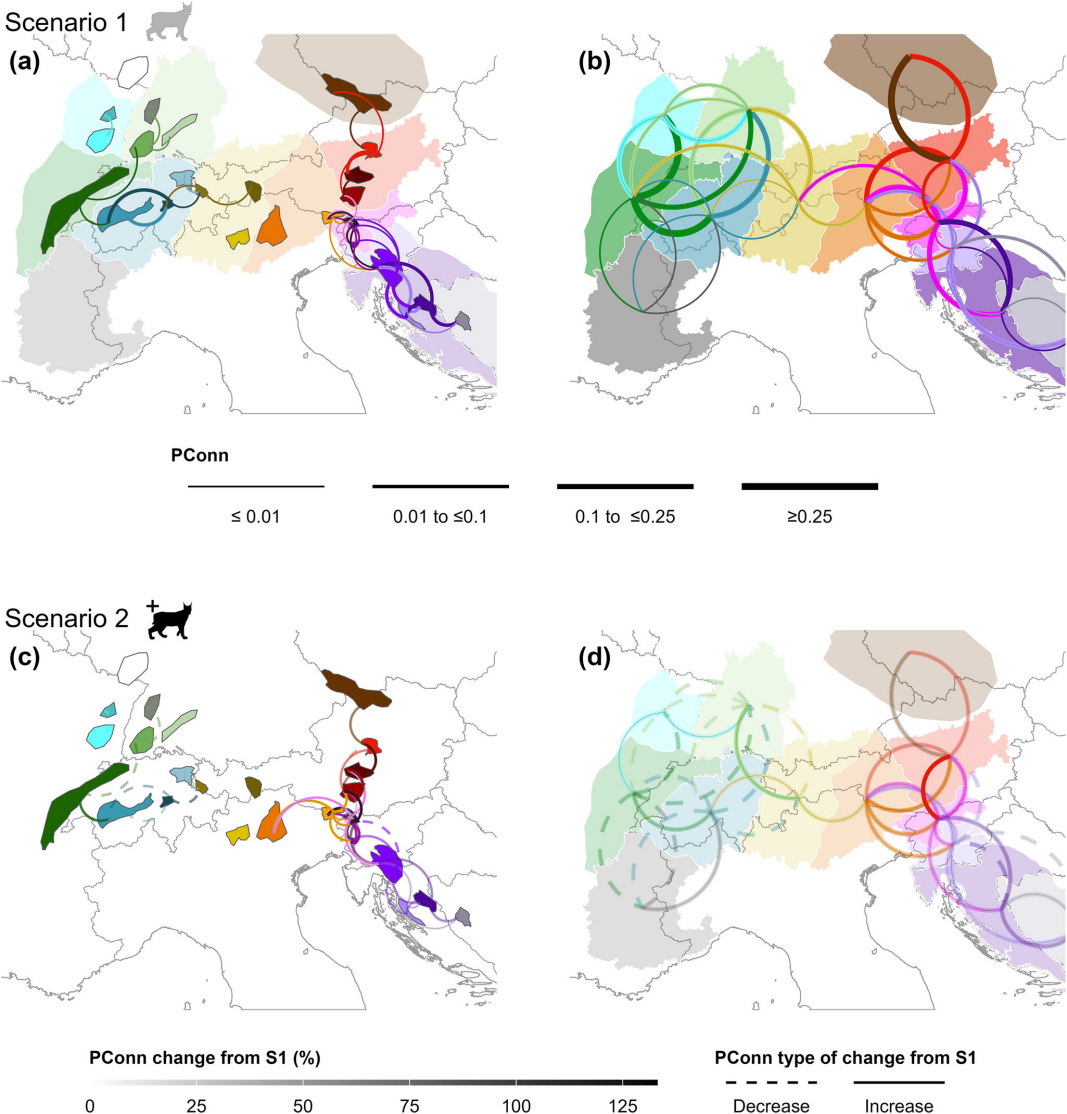
Connectivity network from 2019 to 2040 in simulation (a, b) without population reinforcements (scenario 1) and in simulation (c, d) with population reinforcements (scenario 2): (a, c) local connectivity in target patches (linewidths, connectivity probability level *p*_conn_ ≤ 0.01, 0.01 < *p*_conn_ ≤ 0.1, 0.1 < *p*_conn_ ≤ 0.25, *p*_conn_ > 0.25) and (b, d) change in regional connectivity from scenario 1 (transparent lines) and direction of change (dashed lines, connectivity decrease; solid lines, connectivity increase; line color, patch of origin of the immigrant lynx; *P*_conn_, rate of at least one immigration event per year from 2019 to 2040 in the simulations).

Connectivity was highest (*p*_conn_ > 0.25) from the Jura Mountains to the northwestern Alps and Vosges, and from the BBA to Austria (Figure 3). The connectivity within the eastern populations (southeastern Alps, Dinaric, Austrian, and BBA population) was predicted to be better than within the western populations (northwestern Alps, Jura Mountains) (Figure 3b,d). No regional patch was completely isolated.

As expected, connectivity between target patches was much lower than for regional patches (Figure 3), but all target patches in the southeastern Alps were connected with the Dinaric core area (Figure 3a,c). In the release and projection period in scenario 2, the connectivity with neighboring target patches in the eastern Alps was improved, with six additional connectivity paths (Figure 3a,c). The highest number of connectivity paths was among target patches within the southeastern Alps, with a total of 16 connectivity paths, being also the area with the highest increase in connectivity compared with scenario 1. This makes the areas between the Dinarics and the Austrian Als a hotspot for connectivity, promoting connectivity to neighbouring areas like the Italian southeastern Alps (Figure 3). But target patch connectivity was overall low, with an average connectivity rate of *p*_conn_ = 0.01, which means that from the 100 repetitions for a year in our simulation, there is one when a lynx born in patch *x* arrives at patch *y*. This translates to a 1% probability of having at least one successful immigration per year in the real world. On average, two out of three target patches in the central Alps remained isolated with no immigration (Figure 3a,c).

The patches with the highest *p*_conn_ from a surrounding patch in scenario 2 were in the Dinaric (patch 2) and the northwestern alpine (patch 7) and in the Austrian Alps (patch 25) populations (*p*_conn_ 0.01< to ≤ 0.1). In the core area of the Dinaric population (patch 2), individuals arrived mostly from patches 10 and 11 in Croatia and patch 23 in the southeastern Alps. In the northwestern alpine population (patch 7), most of the lynx that arrived were from other target patches in the northwestern Alps (e.g., patch 6), and fewer from the Jura Mts (patch 8). Connectivity between target patches in core areas of lynx populations and the rest of the patches did not change considerably between scenarios and remained very low (*p*_conn_ = 0.01) (Figure 3a,c).

**FIGURE 4:**
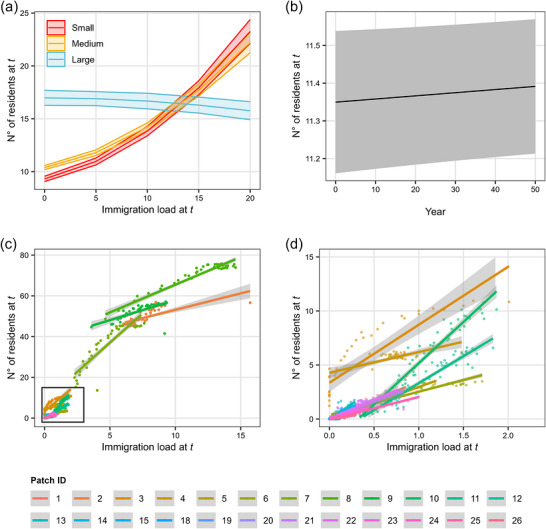
Effects and standard error (SE) of (a) immigration load and patch size (small, 113 km^2^; medium, 1062 km^2^; large, 6900 km^2^), (b) year on the population size of lynx residents in the target patches at timestep *t*+1 (y‐axis), (c) effect of the immigration load at year *t* on the resident's population size at *t*+1 for each target patch, and (d) subset from (c).

The resident population size was positively associated with immigration load (β [SE] = 49.2446 [4.586], *p* < 0.001) (Appendix S12) for small (≤113 km^2^) and medium‐sized (113−1062 km^2^) patches, although it became insignificant for large patches (1062−6900 km^2^) (β = −0.001 [0.0001], *p* < 0.001) (Figure 4 & Appendix S12). The rest of the explanatory variables (meanRes, area), except for year, were also significant (*p* < 0.001) but with small coefficient values (β −0.0076 to 0.001). Therefore, they were not biologically relevant (Appendix S12).

### Minimum release population

**FIGURE 5:**
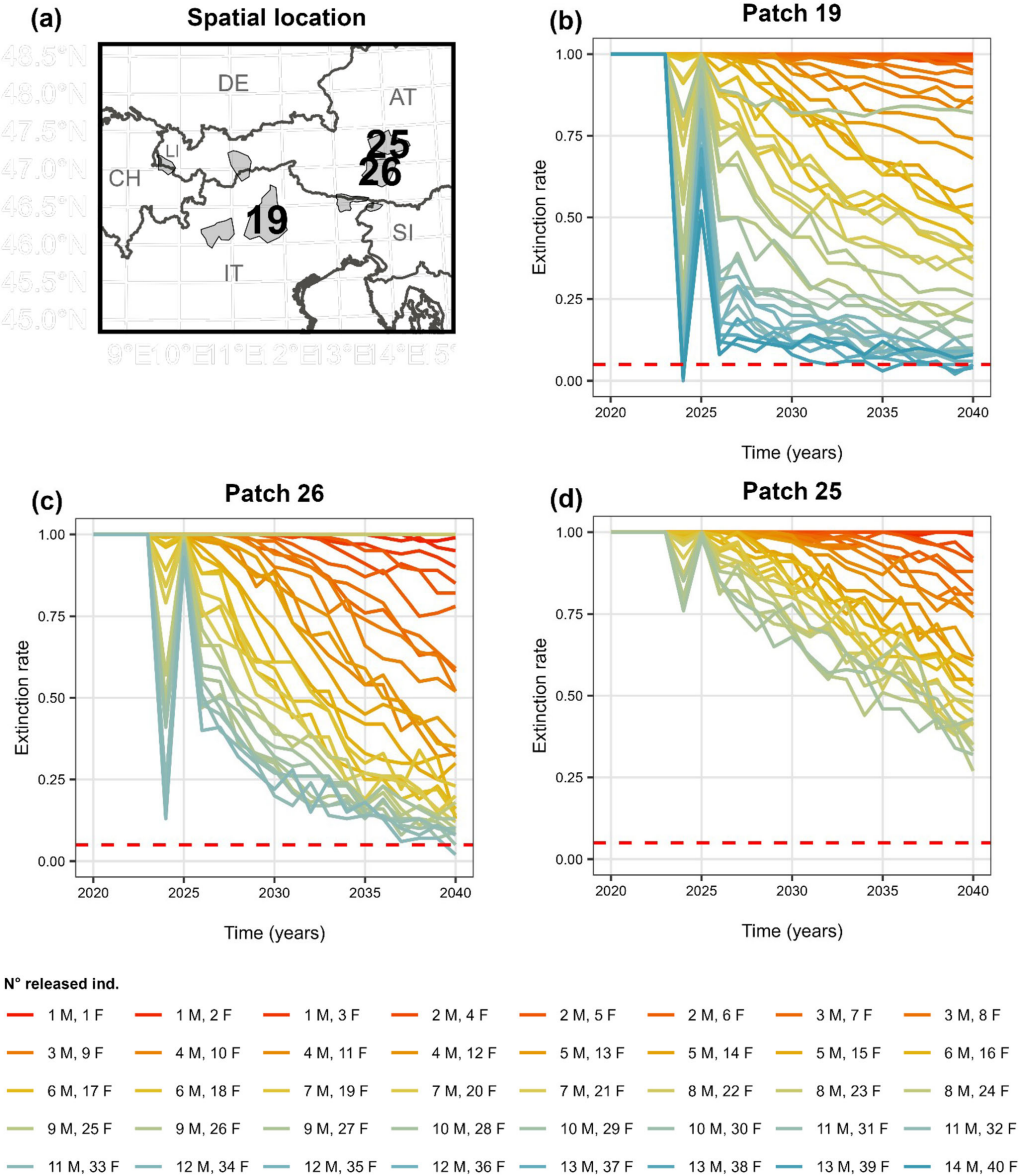
(a) Location of target patches in the eastern Alps and minimum lynx release population for a future viable population in target patches (b) 19, (c) 26, and (d) 25 (population viability threshold, 20; red dashed line, extinction threshold of 5%).

The model predicted that the most suitable target patches to create a stepping‐stone population, fulfilling our criteria with *p*_ext_ < 0.05 and at least 20 individuals by the end of the simulation, were patches 19 and 26 in the Austrian and Italian eastern Alps, respectively (Figure 5a), which are also the biggest patches. The MRP was of 13 males and 37 females in patch 19, and 11 males and 33 females in patch 26 (Figure 5b,c). In patch 25, the release of 10 males and 28 females was not enough to maintain a lynx population with *p*_ext_ < 0.05 (*p*_ext_ = 0.32) (Figure 5d). In all the other patches, *p*_ext_ exceeded 0.5 at the end of the simulation.

## DISCUSSION

The SE‐IBM are an excellent tool to model possible population trends because they account for the animals’ life history and the landscape they move through and inhabit. This can improve management, especially when comparing the outcome of different management scenarios. We used the lynx as a model species and analyzed the viability and connectivity of six lynx populations in western and central Europe, and estimated the MRP needed to establish a stepping‐stone population. Specifically, we compared the predicted development of two populations with and without reinforcement, and whether reinforcements improved connectivity with neighboring populations.

Between 2021 and 2023, the southeastern alpine lynx population was reinforced with 11 individuals. Based on the model forecast, this management measure saved the southeastern alpine population from extinction. At the end of the simulations, in 2040, this population was predicted to range from 20 to 40 resident individuals, with an increasing trend. In contrast, the model did not detect any demographic effect of the reinforcement of the Dinaric population with 12 translocated lynx, and the population remained stable in the model. However, if the consequences of low genetic diversity on lynx survival or reproduction would influence the model outcome, we would expect a demographic improvement in the population after the increase of genetic variation through the lynx translocations. Although translocations are not predicted to affect the population trend in the Dinaric population in our model, they improved the connectivity in the southeastern Alpine population, increasing its viability. The BBA and Jura Mountains populations also remained stable in our model, although the causes of stagnation most likely differ. In the model, the size of the BBA population most likely remained constant due to high added mortality (Heurich et al., 2018), whereas the Jura population most likely lacked habitat for territory settlement. The low added mortality rates in the Jura population and higher connectivity to neighboring areas compared with other populations suggest all available territories were occupied. In contrast, the population size increase in the northwestern Alps and Austria was likely due to a combination of habitat availability for territory settlement and a relatively low added mortality, allowing these populations to expand.

### Prospects of lynx populations in central Europe

Lynx translocations proved beneficial for population growth in the small southeastern alpine population. However, even though the southeastern Alpine and Dinaric populations have higher connectivity probabilities compared with other populations, the number of individuals in both populations did not reach the criteria of the 50−500 rule (Franklin, 1980) until 2040. Ideally, the northwestern alpine and Austrian populations would expand toward the southeastern Alps, thus establishing effective connectivity. Our results showed this is unlikely to happen soon. Linear infrastructures represent a major impediment. For example, connectivity between the Dinaric and southeastern alpine population is currently limited by the Ljubljana‐Koper highway (Krofel et al., 2006). We found that connectivity between eastern (BBA, Austria, southeastern Alps, and Dinaric Mountains) and western (Jura Mountains, northwestern Alps) populations is unlikely to occur in the midterm (∼20 years) without the creation of further stepping‐stone populations despite medium‐high landscape connectivity (Iannella et al., 2024). Connectivity to adjacent populations may be possible with periodic reintroductions in a stepping‐stone fashion (Morris et al., 2021). With current survival and reproductive rates, the creation of a stepping‐stone population in the eastern Alps could be achieved with the release of 45−50 lynx as a very conservative measure, assuming there is negligible connectivity, in patch 19 (northeastern Italy), and patch 26 (Carinthia, Austria).

The historic public acceptance level in Austria is very low for large carnivores (Zeiler et al., 1999), which can result in unsustainable levels of illegal killings (Kaczensky et al., 2011). Releasing such numbers of animals in 1 year is unusual and difficult in translocation projects that tend to extend over 3−5 years; hence, other strategies may be explored. Applied to a population, adding new individuals may partially compensate in the short term for the ones lost, particularly from a genetic perspective.

Population‐level benefits of translocations will probably be greater in reality than in our model; adding new individuals into an inbred population is expected to increase the genetic pool and decrease inbreeding (Weeks et al., 2011). This was already observed in the Dinaric and southeastern alpine lynx populations, where the genetic structure of the population improved after translocations (Fležar et al., 2023; Krofel et al., 2025). The increase of genetic diversity can have positive impacts on reproduction and survival rates that the model is not able to capture, hence underestimating the demographic improvement in the population. Such improvements have already been seen in the Dinaric population (Keller & Waller, 2002; Skrbinšek et al., 2019). Demographic improvement following translocations has also been observed in the Florida panther (*Puma concolor coryi*) (Johnson et al., 2010) and bighorn sheep (*Ovis canadensis*) (Hogg et al., 2006) in the United States and in the mountain pygmy possum (*Burramy parvus*) in Australia (Weeks et al., 2017).

### Model limitations and assumptions

A common limitation of IBMs is the uncertainty in parameter estimation and the need for retrospective calibration (Railsback & Grimm, 2019), especially in the case of nonlinear dynamics (Bennett, 2002). This “inverse fitting” limits uncertainties in the parameter space and serves to evaluate the model and predictions (Bennett, 2002). To further reduce parameter uncertainties, we used available data whenever possible. We used known reproductive parameters and estimated mortality parameters obtained from multiple lynx populations (Appendix S2). Additional mortality was the only unknown parameter in our model and was inversely fitted for each population to regulate the excess of individuals when baseline and traffic mortality were not enough. Overall, annual survival rates in central Europe in the reference period (1995−2018) in our model ranged from 0.46 to 0.65 for kittens and dispersing lynx combined, and from 0.65 to 0.82 for resident lynx (Appendix S11). Our survival rates are consistent with previous simulation studies (Heurich et al., 2018) and similar to results from field studies for most of the populations (Appendix S11). However, the simulated survival rates for the Dinaric and southeastern alpine lynx populations are lower than those from (Premier et al. 2025) that are solely based on telemetry data. This is likely because the added mortality in our model accounts for the negative effects of inbreeding in reproduction and survival of lynx, which may not be captured by telemetry data.

Moreover, the dispersal submodel was calibrated with data from the 1990s in the Jura Mountains, with the longest dispersal distance being <100 km, while in the meantime, dispersal of twice the distance has been observed in western and central Europe (Bonn Lynx Expert Group, 2021; Drouet‐Hoguet et al., 2021). Finally, we assumed a static landscape and environmental stochasticity over time, which could create a mismatch with the current landscape in the form of future habitat degradation or improvement, which would impact connectivity and population viability.

### Implications for conservation

Lynx populations in western and central Europe are slowly growing or demographically stable, but the lack of connectivity between these populations raises the question of whether these will survive in the long‐term without active management. Hence, transboundary coordinated monitoring plans (demographic and genetic) and actions actively involving all stakeholders is paramount to restore a favorable conservation status for the species. Despite low connectivity between populations, habitat quality and landscape connectivity are overall acceptable (Iannella et al., 2024), threats like anthropogenic mortality and landscape fragmentation, which can slow down population growth and hamper dispersal (Heurich et al., 2018; Vogt et al., 2025), must be addressed. Wildlife passages are partially compensatory to habitat fragmentation, and conservation measures should focus on protecting and rehabilitating lynx habitat and dispersal corridors (Iannella et al., 2024) to conserve the populations and achieve population connectivity through stepping‐stones. Although we may not achieve our objective of establishing population connectivity between lynx populations in western and central Europe within one reintroduction project, it might be feasible with the combined efforts of multiple transboundary projects. A current example is the recent collaboration among lynx conservationists within the realm of the Carpathian lynx metapopulation (Linking Lynx, 2024).

Our work underlines the potential of SE‐IBMs to simulate population dynamics on a large scale and to serve as an a priori analysis and framework to plan translocations and management interventions and assess their success at a continental scale. It also provides evidence on the effectiveness of the creation of lynx stepping‐stones to improve population connectivity. However, a deep understanding of the effect of stepping‐stones on surrounding populations in terms of connectivity is lacking. Hence, it is necessary to focus future research on the effects of creating stepping‐stones on lynx population demography and connectivity to ensure the optimal result for lynx conservation. We recommend exploring the effect of the frequency of reinforcements and sex ratio on conservation success of stepping‐stones, as well as exploring the effects of variation of survival and dispersal rates between lynx populations, the identification of major barriers for dispersal, and the incorporation of genetic diversity in the model.

## AUTHOR CONTRIBUTIONS

E.S.A., A.P., S.K.‐S., and A.M.‐J. designed the study. E.B., L.B., R.C., U.F., T.G., M.K., T.M., H.P., M.S., I.T., K.V., and F.Z. provided field data. E.S.A. led the analysis of the data and model simulations with support from A.P., J.P., S.K.‐S., and A.M.‐J. E.S.A. led the interpretation of the results and the writing of the manuscript. A.P., J.P., T.K., S.K.‐S., and A.M.‐J. supported the interpretation of the results. A.P., J.P., T.K., E.B., C.B.‐W., L.B., R.C., T.E., U.F., T.G., M.K., T.M., P.M., S.P., K.P., H.P., M.S., I.T., K.V., J.V., S.W., F.Z., M.H., S.K.‐S., and A.M.‐J. critically revised the manuscript. M.H., S.K.‐S., and A.M.‐J. supervised the work.

## Supporting information



Supporting Information

## Data Availability

Data are available from the Zenodo Repository at https://doi.org/10.5281/zenodo.19921537. https://datadryad.org/stash/share/t4ms7dAQA6fYBKb5nXPTBY_fBD1KK‐Rj82pW8ehg5i0
