# Supplementary material for: Translocation effects on regional and local population viability and connectivity
Source: Conserv Biol. 2026 May 12;40(4):e70321. doi: 10.1111/cobi.70321 (PMC13392802; doi:10.1111/cobi.70321)
Supplement: Supplementary file 1 — Supporting Information [file COBI-40-e70321-s001.docx]

Translocations support local populations but have limited effect on regional population viability and connectivity in a large carnivore

**Appendices**

**Appendix 1 Methods: S1 – S8**

**Appendix 2 Results: S9 - S12**

**Appendix 3 ODD protocol and S13 - S14**

**Appendix 1. Methods**

In this section we provide supporting information on the study species and methodology.

**Study species**

The Eurasian lynx is a solitary and territorial felid (Breitenmoser & Haller 1993). In central Europe, it is strongly linked to forest habitat (Breitenmoser 1998) and is an ambush predator of ungulates, with preference for roe deer (*Capreolus capreolus*) (Breitenmoser & Haller 1993; Breitenmoser 1998; Jobin et al. 2000; Zimmermann et al. 2007; Molinari-Jobin et al. 2007; Krofel et al. 2011). Reproduction is restricted to residents (territory holders), which mate between February and mid-April, and females give birth usually in late May to 1-4 kittens (Breitenmoser et al. 2000; Gaillard et al. 2014). The female takes care of the kittens alone until their dispersal at the age of about 10 months (Breitenmoser et al. 2000).

**
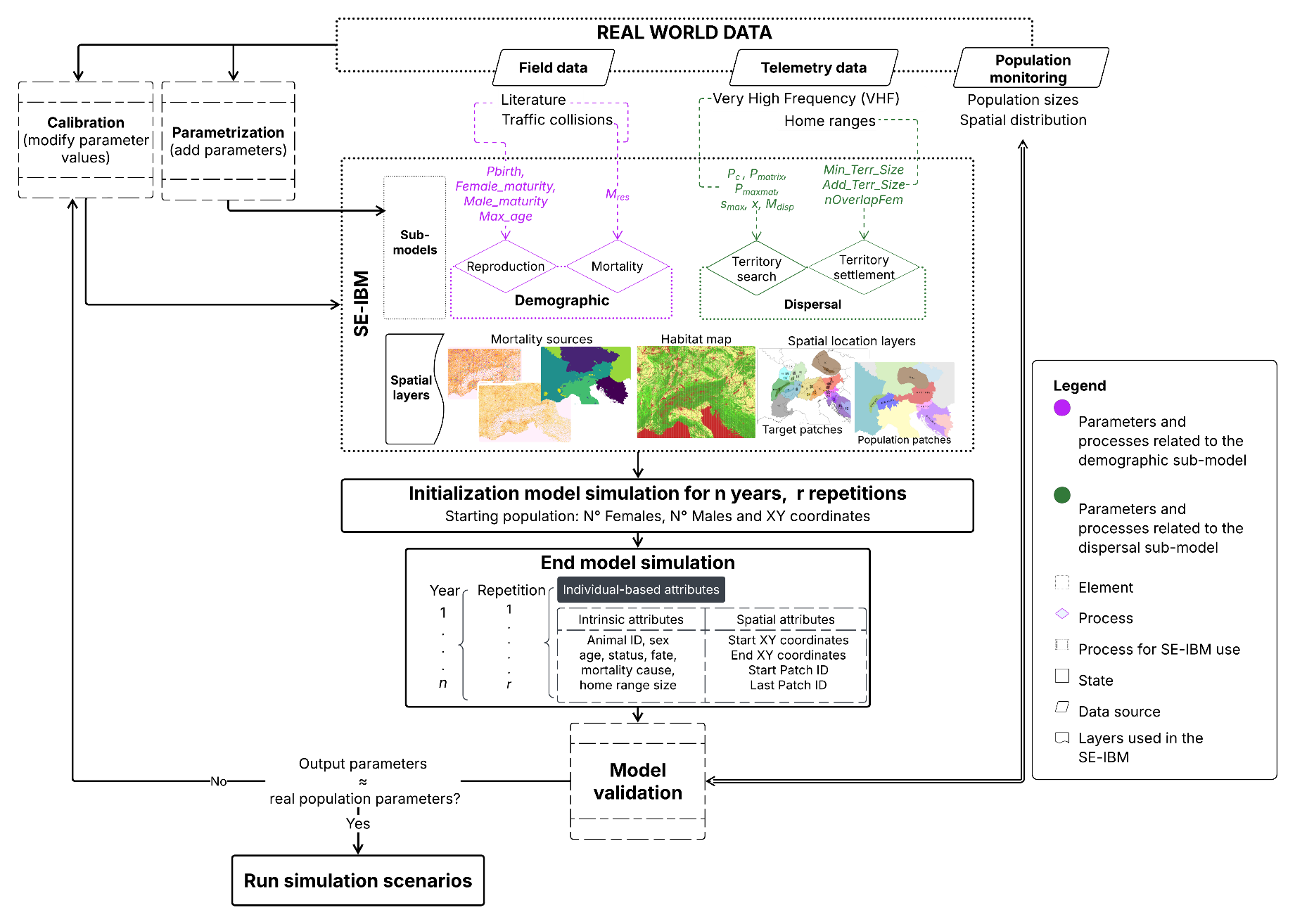
Modelling process**

**Appendix S1** Description of the modelling process including data sources, model structure, and model processes from model parametrization and calibration to model validation and posterior simulation of scenarios. Arrows indicate flow of information. Diagonal boxes indicate data, diamonds indicate processes. Long-dashed boxes indicate mandatory processes (parametrization, calibration and validation). Pink elements relate to the demographic sub-model and green elements relate to the dispersal submodel.

**Model parametrization and calibration**

The dispersal sub-model (parameters Autocorrelation, Pmatrix, Maxmatrixvalue, smax*, x* and Mdisp*,* Appendix S2) was initially parametrized and calibrated with telemetry data collected from 1988 to 1991 of dispersing lynx in the Jura Mts. (Breitenmoser et. al. 1993 as cited in Kramer-Schadt et al., 2004). Demographic parameters of litter size (Litter_size), juvenile survival (Juv_survival), female and male year of maturity (Female_maturity, Male_maturity), and maximum age a lynx can survive in the model (Max_age), were extracted from field studies (Appendix S2, Kramer-Schadt et al. 2005). The rest of the parameters (demographic sub-model and corrector factors, Appendix S2) were calibrated for the purpose of this study. Demographic parameters of reproduction and territory size (Pbirth, MinTerrSize, AddTerrSize, CorrFactRes, Add_mort, CorrFactDisp, Appendix S2) were calculated from field data for the purpose of this study. Parameters of survival and natural and road mortality were inversely fitted to yield mortality rates from field reports (see Heurich et al. 2018 for details). Added mortality parameter was fitted in the model to regulate the surplus of individuals in a population to match real population sizes in 1995 (Appendix S2).

The rate of reproduction (Pbirth) corresponds to the average of the combined reproduction rate of the Swiss Alpine lynx population (Bauduin et al. 2021) and the BBA population (Mináriková et al. 2023; Wölfl et al. 2023). Female territory size is calculated as the number of breeding cells in the non-overlapping area of female home ranges (territory) (Appendix S3). We used 35 female home ranges calculated as a 95% Minimum Convex Polygon (95% MCP) from five populations (Appendix S3).

**Appendix S2** Input parameters and their symbols of the simulation model for the expansion phase 1 (1970–1995/1996) and phase 2 (1996/97–2040) of the simulation model of both demographic and dispersal sub-models. Populations are specified when the input parameters varied between the populations. Parameters in bold were calibrated for the purpose of this study. The parameter Juv_survival is included within the litter size, thus is 1. Correction factors for roads of residents (CorrFactRes) and dispersers (CorrFactDisp) and added mortality (Add_mort) parameters were inversely fitted in the model during the calibration of the model.

| **Parameter** | **Symbol** | **Population** | **Input value** | | | | |  |
| --- | --- | --- | --- | --- | --- | --- | --- | --- |
|  |  |  | **Phase 1** | **Phase 2** | | | |  |
| Demographic sub-model | | | | |  | | | |
| Birth probability | Pbirth^a^ |  | 0.83^b^ | 0.83^b^ | | | |  |
| Mean litter size | Litter_size |  | 1.5 ^c,d^ | 1.5 ^c,d^ | | | |  |
| Juvenile survival | Juv_survival |  | 1 | 1 | | | |  |
| Age of sexual maturity (year) | Female _maturity / Male_maturity |  | 2^e^ | 2^e^ | | | |  |
| Maximum age (years) | Max_age |  | 15 | 15 | | | |  |
| Minimum territory size | MinTerrSize^a^ |  | 42^b^ | 42^b^ | | | |  |
| Added territory size | AddTerrSize^a^ |  | 63^b^ | 63^b^ | | | |  |
| Maximum number of female territories overlapped by one male | Max_n_female_overlap_per_male |  | 3 ^c,f^ | 3 ^c,f^ | | | |  |
| Annual baseline mortality of residents | Mres |  | 0.1^c,f^ | 0.1 ^c,f^ | | | |  |
| Traffic mortality of residents | CorrFactRes^a^ |  | 10000 | 10000 | | | |  |
| Additional mortality | Add_mort^a^ | Jura Mts. | 0.09 | 0.19 | | | |  |
|  |  | Swiss Alps | 0.09 | 0.19 | | | |  |
|  |  | Austria | 0.24 | 0.18 | | | |  |
|  |  | BBA | 0.20 | 0.27 | | | |  |
|  |  | Dinaric | 0.25 | 0.29 | | | |  |
|  |  | SE Alps | 0.25 | 0.29 | | | |  |
| Dispersal sub-model | | | | | | |  |  |
| Correlation factor | Autocorrelation |  | 0.5 ^b,c,f^ | 0.5^b,c,f^ | | | |  |
| Probability of stepping into matrix habitat cell | Pmatrix |  | 0.03^b,c,f^ | 0.03 ^b,c,f^ | | | |  |
| Maximum number of steps in matrix habitat before returning | Pmaxmatrix |  | 10 ^b,c,f^ | 10 ^b,c,f^ | | | |  |
| Maximum number of steps per day | smax |  | 45^f^ | 45^f^ | | | |  |
| Exponent step distribution | *x* |  | 11^f^ | 11^f^ | | | |  |
| Daily mortality probability of dispersers | Mdisp |  | 0.0006^g^ | 0.0006^g^ | | | |  |
| Mortality of dispersers due to linear features | CorrFactDisp^a^ | Jura Mts. | 2000000 | 1400000 | | | |  |
|  |  | Swiss Alps | 2000000 | 1400000 | | | |  |
|  |  | Austria | 1800000 | 1400000 | | | |  |
|  |  | BBA | 2800000 | 1400000 | | | |  |
|  |  | Dinaric | 80000000 | 1400000 | | | |  |
| ^a^Parameter calculated for the purpose of this study  ^b^Parameter calculated from field data  ^c^Breitenmoser & Haller (1993)  ^d^Breitenmoser et al. (2000)  ^e^Jewgenow et al. (2014)  ^f^Kramer-Schadt et al. (2004)  ^g^Gaona et al. (1998) | | | | | |  |  |  |

**Mortality and other parameters**

Mortality is introduced in the model through different sources. Baseline mortality parameters (Mdisp and Mres, Appendix S2) represent all the mortality that is not due to traffic, hunting, illegal killing or unexplained mortality, i.e., diseases, old age. Residents are assigned a yearly baseline mortality probability of 0.1, translated into ~10% yearly baseline mortality rate as reported in the Swiss Jura (Breitenmoser-Würsten et al. 2007). Dispersers have a daily baseline mortality probability of 0.0006, which is translated into ~22% yearly baseline mortality (Breitenmoser-Würsten et al. 2007). Mortality by linear features (roads and rivers) complements baseline mortality and is introduced through linear feature maps (Appendix S4b,c), which are regulated with correction factors to yield the reported number of dead lynx by traffic collision in the Jura Mts. and Swiss Alps (KORA, unpublished), and BBA (Mináriková et al. 2023; Wölfl et al. 2023).

**Home range size of female lynx**

We used 36 home ranges from 30 females (6 females were monitored twice and the respective HR was calculated for each monitoring period; Appendix S3) in Central Europe calculated from field data with 95% Minimum Convex Polygons (95% MCP). We used Voronoi tessellations to calculate the territory size of females with spatio-temporally overlapping home ranges and counted the number of breeding cells within each territory. Home ranges that did not overlap with any other were considered to be a territory. The average number of breeding cells (mean = 105) and the standard deviation (SD = 63) across all territories define the minimum territory size (MinTerrSize, Appendix S2) and maximum territory size a female can have in the model. In this line, the minimum territory size is the average minus one standard deviation (MinTerrSize = 42 cells). A number of additional breeding cells (AddedTerrSize, Appendix S2), ranging between 0 and the standard deviation (SD = 63), is drawn from a uniform distribution and added to the minimum territory size to define the territory size each female will hold. Thus, the territory size of a female is 42 + range (0-63) breeding cells. Males overlap their territories with 1-3 females, thus their territory size indirectly emerges to be larger than the females’.

**Appendix S3** Home Range (HR) estimations through Minimum Convex Polygons (MCPs) with 95% CI of female lynx used to estimate the territory sizes. The table describes the population of each female, their ID, the start and end of the monitoring period and the estimated HR area in km^2^.

| Population | Female ID | depl_date  (dd.mm.yyyy) | end_date  (dd.mm.yyyy) | HR area (km^2^) |
| --- | --- | --- | --- | --- |
| Western Alps | Rika | 19.04.2012 | 29.10.2012 | 185.669 |
| Western Alps | Mari | 08.03.2011 | 21.11.2011 | 112.216 |
| Western Alps | Eywa | 27.12.2013 | 10.12.2014 | 70.99 |
| Western Alps | Suna | 06.01.2013 | 16.08.2013 | 96.96 |
| Western Alps | Mila | 24.10.2013 | 08.05.2014 | 261.032 |
| Western Alps | Isis | 07.03.2014 | 13.01.2015 | 82.5 |
| Western Alps | Kana | 01.12.2012 | 29.05.2013 | 52.466 |
| Western Alps | Lela | 17.03.2016 | 29.12.2016 | 140.798 |
| Western Alps | Isis1 | 09.12.2016 | 03.04.2018 | 126.6 |
| Western Alps | Lyra | 27.11.2016 | 02.01.2018 | 78.187 |
| Western Alps | Wega | 07.12.2016 | 17.10.2017 | 89.183 |
| Western Alps | Neve | 17.03.2016 | 03.04.2017 | 67.788 |
| Western Alps | Cara | 29.03.2016 | 13.11.2016 | 117.752 |
| Western Alps | Eywa2 | - | - | 65.568 |
| Austria | Aira | 17.03.2017 | 31.12.2017 | 186.327 |
| Austria | Aira | 01.01.2018 | 31.12.2018 | 197.547 |
| Austria | Freia | 09.05.2011 | 31.12.2012 | 218.713 |
| Austria | Freia | 01.01.2012 | 31.12.2013 | 162.558 |
| Austria | Kora | 25.03.2013 | 31.12.2013 | 55.378 |
| Austria | Kora | 01.01.2014 | 31.12.2014 | 28.714 |
| Austria | Skadi | 09.03.2015 | 31.12.2015 | 163.787 |
| Dinaric | Dina | 29.12.2006 | 02.09.2007 | 534 |
| Dinaric | Dina2 | 01.01.2007 | 31.12.2007 | 100 |
| Dinaric | Maja | 28.01.2012 | 10.07.2012 | 54 |
| Dinaric | Petra | 01.03.2021 | 11.01.2022 | 90 |
| Dinaric | Snezka | 10.02.2008 | 04.10.2008 | 90 |
| SE Alps | Aida | 26.04.2021 | 11.01.2022 | 40 |
| SE Alps | Julija | 28.04.2021 | 11.01.2022 | 80 |
| SE Alps | Lenka | 28.04.2021 | 11.01.2022 | 140 |
| BBA | F1 | 03.11.1999 | 01.06.2000 | 306.14 |
| BBA | F2 | 12.29.2000 | 28.06.2002 | 149.49 |
| BBA | F4 | 22.05.2002 | 28.05.2004 | 291.5 |
| BBA | F6 | 28.02.2011 | 13.03.2012 | 96.1 |
| BBA | F7 | 17.01.2007 | 12.03.2008 | 173.63 |
| BBA | F8 | 17.03.2010 | 31.10.2011 | 148.16 |
| BBA | F10 | 01.05.2011 | 27.11.2012 | 92.47 |

**Maps of the model**

The model is composed of several hierarchical spatial raster layers. The habitat map (Appendix S4a) is the most important one where spatial processes of dispersal and territory establishment occur. The habitat is classified into four categories following the description in Schadt et al. (2002) and to which dispersers react: non-suitable habitat for lynx (barrier), habitat occasionally used by lynx (matrix), preferred habitat for travelling (dispersal), and habitat suitable for setting up a territory (breeding), based on the lynx’ perception (Schadt et al. 2002a). It is complemented with two linear features grid maps: a) a density risk map for residents’ mortality (Appendix S4b); and b) spatially explicit mortality map for dispersers (Appendix S4c). The density risk map (b) does not include rivers and assigns a mortality to residents based on the road density within the territory. The impact of mortality by linear features in both maps is regulated through a correction factor (Schadt et al. 2002a) (Appendix S2). The explicit mortality by linear features map (a) includes roads (primary, secondary and tertiary), motorways and rivers. Dispersers that step into a raster cell with any of these features are assigned a mortality probability according to each class. An added mortality map (Appendix S4d) accounts for other sources of mortality such as poaching or decreased survival by inbreeding, excluding natural and traffic mortality, and varies within the study area. National parks are assigned half of the added mortality of the country where they are located as we assume there is a certain level of protection against illegal killing (Müller et al. 2014; Magg et al. 2015). Lynx populations are spatially defined in the model (Appendix S4e). In addition, a connectivity and an emigration map are used in the model but do not interfere in any demographic processes. The connectivity map is composed of 26 local patches (Appendix S4f) surrounded by the regional patches (Appendix S4f) which function is to track the movement of individuals, recording lynx when they enter or leave a patch. The emigration map delineated the study area in the model, when an individual leaves the study area it is counted as “emigrated” and is not considered to return, being excluded of the annual mortality statistics. Nevertheless, emigrant lynx are rare in our study as the study area is large enough to cover the area of interest.

The landcover data was obtained from the Corine Land Cover (CLC) map 2018 v.20 Service (European Topic Centre on Land Cover & Environment Satellite Data Centre 2018) which has a resolution of 100 meters. The linear features datafiles were downloaded as shapefiles from the Geofabrik Download Server from (Open Street Map 2020). Shapefiles of national parks were obtained from the Protected Planet WDPA dataset (UNEP-WCMC and IUCN 2020).


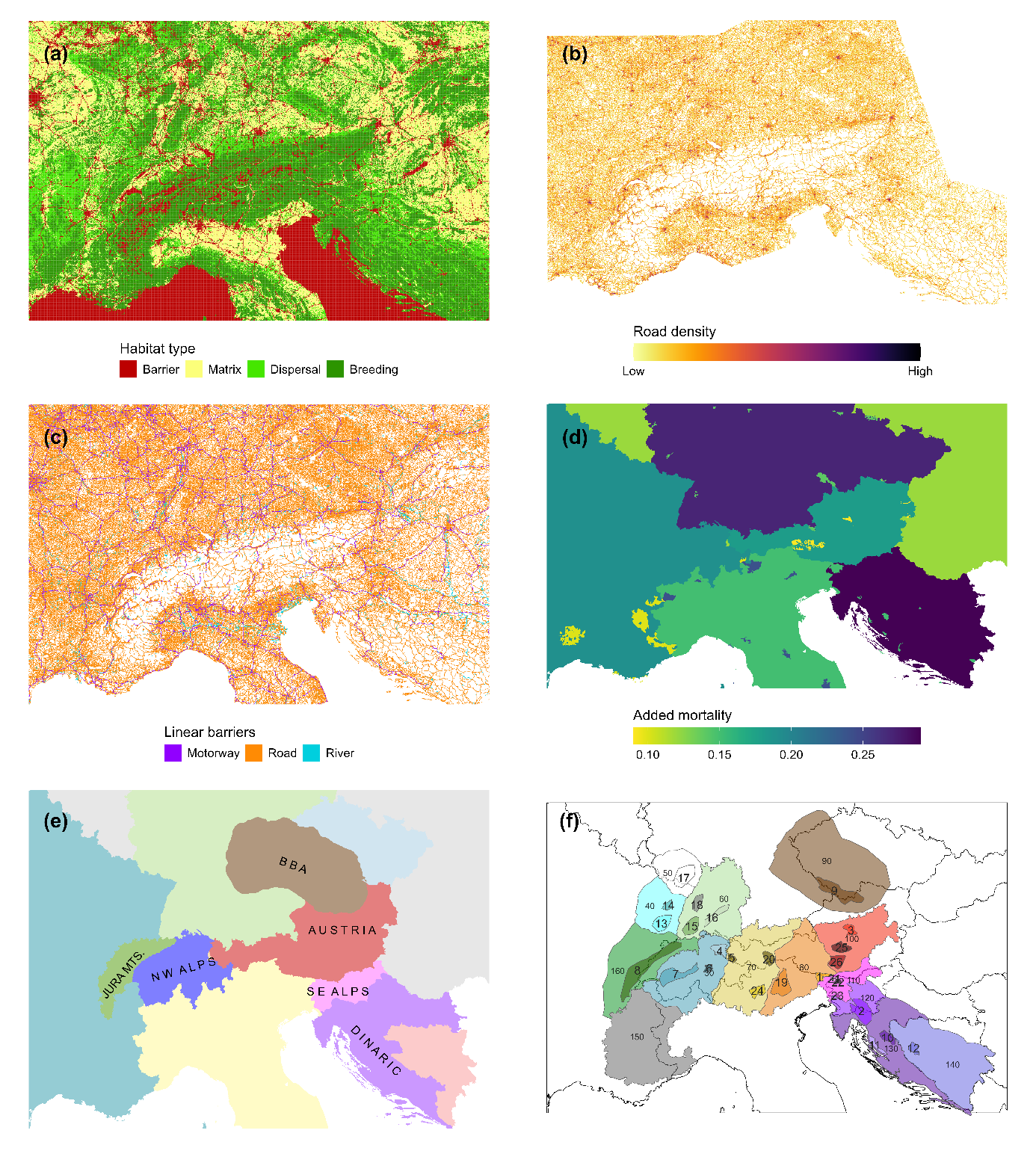
**Appendix S4** Spatial layers used in the model. (a) Layers include the lynx habitat map where the movement decision-making process takes place; (b) linear density and (c), linear explicit features introducing mortality in addition to (d) an added mortality map. A (e) lynx population map and a (f) patch connectivity map classify the landscape into lynx populations and patches of conservation interest for lynx, respectively. In the patch connectivity map (f) we distinguish large regional patches (light colour), classifying the landscape in larger geographic areas (e.g. south western Alps, patch ID =150), and smaller target patches (darker, with numbered ID 1 to 26). Target patches are inside regional patches to capure population dynamics at specific locations.

Habitat map

The habitat map (Appendix S4a) is based on the Corine Land Cover (CLC) raster land classification from 2018 v.20 Service (European Tropic Centre on Land Cover & Environment Satellite Data Centre, 2018). The CLC map has a cell resolution of 100 meters and classifies the land use into 26 categories. For our purpose we increased the resolution to 1 km^2^ and re-classified the habitat categories into four habitat types defined by a habitat suitability value (HS) ranging from 0 to 4. Barriers (HS = 0) are non-suitable habitat, avoided by the lynx, i.e., human infrastructures, water bodies and motorways. Matrix habitat (HS = 2), is non-preferred habitat but occasionally used for dispersal i.e., agricultural land. Dispersal habitat (HS = 3) are forest patches less than 1 km^2^ apart and used for dispersal and travelling. Breeding habitat (HS = 4), suitable for territory establishment and defined as continuous forest habitat large enough to sustain a territory, defined by the parameter MinTerrSize and AddedTerrSize (Appendix S2) according to the minimum territory size. The difference between dispersal and breeding habitat depends on whether the area is large enough to hold a territory. Hence, breeding habitat emerges from dispersal habitat. Breeding habitat is hence identified as continuous areas of forest and semi-natural habitat (i.e. meadows), below 2,500 meters a.s.l. (AMJ, pers. comm. February 2020). It is calculated via a moving window over 3x3 neighboring raster cells, and those focal cells with at least 3 contiguous cells with forest habitat were classified as breeding habitat for the lynx. The altitude threshold was chosen under the assumption that lynx in central Europe choose lower altitudes to breed (AMJ, pers. comm. February 2020). The habitat map was checked by experts in lynx (Dr. Anja Molinari-Jobin), compared with those of previous studies (Becker 2013; Magg et al. 2015; Heurich et al. 2018), and validated by overlaying home ranges of resident females to confirm lynx habitat selection. Topographic data from the study area was obtained as Digital Elevation Models (DEM) in 25 x 25 m resolution, EU-DEM version1.1 from Copernicus Land Monitoring Service (European Topic Centre on Land Cover & Environment Satellite Data Centre 2018).

Linear features map

Linear features (roads and rivers) are represented by two different methods: a) density map (linear mortality for residents) (Appendix S4b) and b) spatially explicit mortality map (linear mortality for dispersers) (Appendix S4c) and. Both maps represent different methods through which mortality is added to the model. The density risk map for residents’ mortality (Appendix S4b) is a proxy for mortality probability based on road density. Rivers are excluded because the model assumes that residents are well aware of their environment and do not cross a river if there is a high risk of mortality.

The spatially-explicit mortality map for dispersers (Appendix S4c) is a gridded map of motorways, main roads (two speed lanes) and rivers, ranked in this order from the highest step mortality probability to the lowest. Thus, motorways dominate the hierarchy with the highest step-mortality probability and rivers the lowest. Each class is represented hierarchically in the map by a cell value: motorways have a value of 100, main roads a value of 170 and rivers have a value of 200. When two or more classes of linear features coincide in one cell, the value of the cell corresponds to the feature with the highest rank. Small roads (i.e., “residential”, “service”, tracks, cycle ways or paths according to the OpenStreetMap classification (Open Street Map 2020)) are not considered since their impact to lynx mortality is considered to be negligible, bridges and tunnels are also excluded. Despite motorways are classified as barrier, this does not exclude the possibility of lynx crossing them. Lynx can cross cells classified as motorways, as cells may be represented in a diagonal array from the lynx position (i.e. directly in front and directly to the side). Thus, mortality probability associated to motorway crossing exists.

Both maps require correction factors to adjust the mortality probability of residents and dispersers according to their respective maps, yielding an annual mortality rate. The correction factors were determined by an inverse fitting process, yielding the average number of dead residents and dispersers in the Jura Mts. and Swiss Alps due to traffic collisions between 1981 and 2019 (unpublished data). Linear feature maps were built using R v4.3.1. (R Core Team 2023) and QGIS Software v.3.6.1 with GRASS v.7.6.1 (QGIS Development Team 2020). Shapefiles of rivers and roads were downloaded from the Geofabrik Download Server from OpenStreetMap (Open Street Map 2020).

**Study Area**

**Appendix S5** Tabular description of the characteristics of the mountain ranges in Western and Central Europe where lynx populations are established described by elevation (metres above sea level -m.a.s.l.-), dominant forest type, percentage of forest cover in the area, average human density per km^2^ or minimum and maximum human density values per km^2^.

| **Study Area** | **Elevation (m.a.s.l.)** | **Forest type** | **% Forest Cover** | **Human Dens**  **(inhabitants/km^2^)** | **References** |
| --- | --- | --- | --- | --- | --- |
| Jura Mts. | 1,700 | Mixed | 50 | 130-140 | Breitenmoser et al. (2007); Gimenez et al. (2019) |
| NW Alps | 4,634 | Mixed | ~33 | 74.6 | Dellagiacoma et al. (2016); Bundesamt für Landestopographie; Bundesamt für Umwelt BAFU |
| BBA | 370 – 1,456 | Mixed |  | 2 - 70 | Heurich et al. (2015); Magg et al. (2016) |
| Dinaric Mts. | 0 - 2,700 | Mixed | 60 | 8 - 28 | Fležar et al. (2023); Güthlin et al.,(2011); Nagel et al., (2017); Zupan Hajna (2019) |
| Kalkalpen  (Austrian Alps) | 385 – 1,963 | Mixed | 81 | <5 - 1168 | (Fuxjäger 2012; Alpine Convention (2015) |
| SE Alps | 0 - 2,864 | Mixed | Unknown but estimated around 60% | 5 - 861 | Alpine Convention (2015; Colucci 2016) |

**Appendix S6** Relation between geographic aera, regional and target patch ID in the patch connectivity layer, and the lynx population from the lynx population layer they represent.

| **Geographic area** | **Regional patch** | **Target Patch** | **Lynx population** |
| --- | --- | --- | --- |
| South Western Alps | 150 | - | - |
| Jura | 160 | 8 | Jura Mts |
| North Western Alps | 30 | 4,6,7 | NW Alps |
| South Vosges | 40 | 13, 14 | - |
| North Vosges | 50 | 17 | - |
| Black Forest | 60 | 15, 16, 18 |  |
| Central Western Alps | 70 | 5, 20, 24 | NW Alps and Austrian |
| Central Eastern Alps | 80 | 19, 1 | Austrian |
| Eastern Alps | 100 | 3, 25, 26 | Austrian |
| Bohemian Forest Ecosystem | 90 | 9 | BBA |
| South Eastern Alps | 110 | 21, 22, 23 | SE Alps |
| Dinarics (Slovenia) | 120 | 2 | Dinarics |
| Dinarics (Croatia) | 130 | 2, 10, 11 | Dinarics |
| Dinarics (Bosnia and Herzegovina) | 140 | 12 | Dinarics |

**Initial conditions of lynx populations in the model and associated target patches**

The initialization of the model requires an initial lynx population. For Phase 1 (1970 – 1995/96) of the simulation, we used the original lynx reintroductions in the 1970’s and 1980’s in Central Europe as well as the subsequent lynx releases for population reinforcement as our starting population. We released in the model the exact number of male and female lynx for each year in the exact location as it occurred in reality (Appendices S7 and S8). At the end of Phase 1 (1995/96), when the model represented the estimated population sizes and population distribution in the field reports, we took a snapshot of the most representative repetition for the simulated year 1995/96 of each reintroduced lynx population and retained the number of residents as well as their sex and xy coordinate in the landscape. We used the population snapshot as the starting population for Phase 2.

**Appendix S7** Starting population size (N) of each simulated lynx population at the beginning of Phase 1 and Phase 2. In Phase 1, number of individuals released corresponds to the initial reintroductions; in Phase 2, number of individuals released corresponds to the median number of resident individuals recorded at the end of phase 1), the years covered in each phase, and target patches associated to each population.

| Population | Phase | Years in phase | N | Target Patches Associated |
| --- | --- | --- | --- | --- |
| Austria | 1 | - | - | 24, 25, 26 |
|  | 2 | 2011 – 2040 | 7 |  |
| BBA | 1 | 1976 – 1996 | 18 | 9 |
|  | 2 | 1997 - 2040 | 69 |  |
| Dinarics | 1 | 1973 – 1995 | 6 | 10, 11, 12 |
|  | 2 | 1996 - 2040 | 97 |  |
| Jura Mts | 1 | 1972 - 1995 | 10 | 7 |
|  | 2 | 1995 - 2040 | 95 |  |
| NW Alps | 1 | 1971 - 1995 | 18 | 4, 5, 6 |
|  | 2 | 1995 - 2040 | 37 |  |
| SE Alps | 1 | 1973 – 1995 | - | 21, 22, 23 |
|  | 2 | 1996 - 2040 | 8 |  |

**Lynx reintroductions**

**Appendix S8** Lynx reintroductions in Central Europe since 1970, lynx population which they belong, site where they took place, number of females (nFem) and males (nMal) released, the fate of the reintroduction and phase of the model where the lynx were simulated. Only the successful reintroductions were taken into account in the model. Those reintroductions within the LIFE Lynx and ULyCA projects are marked with an asterisk (*) and are considered successful.

| **Year** | **Area/Population** | **Site** | **nFem** | **nMal** | **Fate** | **Sim_phase** |
| --- | --- | --- | --- | --- | --- | --- |
| 1970 | BBA | Bavarian Forest | 2 | 2 | Failed |  |
| 1971 | NW-Alps | Grosses Melchtal | 1 | 1 | Success | 1 |
| 1972 | NW-Alps | Chlischlierental | 1 | 1 | Success | 1 |
| 1972 | NW-Alps | Pilatus | 1 | 1 | Success | 1 |
| 1972 | NW-Alps | Engadin | 1 | 1 | Failed | 1 |
| 1972 | Jura | Moutier | 1 | 1 | Success | 1 |
| 1973 | NW-Alps | Pilatus | 1 | 1 | Success | 1 |
| 1973 | Dinaric | Kocevje | 3 | 3 | Success | 1 |
| 1974 | Jura | Creux du Van | 1 | 1 | Success | 1 |
| 1974 | Jura | Vallée de Joux | 2 | 2 | Success | 1 |
| 1975 | NW-Alps | Corbeyrier | 1 | 1 | Success | 1 |
| 1975 | Jura | Creux du Van | 1 | 1 | Success | 1 |
| 1976 | NW-Alps | Grand Muveran | 0 | 2 | Success | 1 |
| 1976 | NW-Alps | Val d'Anniviers | 2 | 2 | Success | 1 |
| 1977 | Austria | Turrach | 2 | 2 | Failed | 1 |
| 1978 | Austria | Turrach | 0 | 2 | Failed | 1 |
| 1979 | Austria | Turrach | 1 | 2 | Failed | 1 |
| 1980 | NW-Alps | Engadin | 1 | 1 | Failed | 1 |
| 1982 | BBA | Stozec | 1 | 1 | Success | 1 |
| 1983 | BBA | Kremelna | 1 | 2 | Success | 1 |
| 1984 | BBA | Strazny | 0 | 1 | Success | 1 |
| 1984 | BBA | Vysoka Myt | 0 | 1 | Success | 1 |
| 1985 | BBA | Horska Kvilda | 1 | 2 | Success | 1 |
| 1987 | BBA | Stozec | 2 | 1 | Success | 1 |
| 1987 | BBA | Obrovec | 1 | 1 | Success | 1 |
| 1989 | NW-Alps | Jorat | 1 | 2 | Success | 1 |
| 1989 | BBA | Horni Kochanov | 1 | 2 | Success | 1 |
| 2001 | W-Alps | Tössstock | 2 | 2 | Success | 2 |
| 2001 | W-Alps | Toggenburg | 1 | 1 | Success | 2 |
| 2003 | NAlps | Ebnet | 0 | 1 | Success | 2 |
| 2003 | NW-Alps | Tössstock | 2 | 0 | Success | 2 |
| 2006 | Jura | Mt Aubert | 0 | 1 | Success | 2 |
| 2007 | Jura | Mt Aubert | 0 | 1 | Success | 2 |
| 2007 | NW-Alps | Toggenburg | 1 | 1 | Success | 2 |
| 2008 | NW-Alps | Rheintal | 1 | 0 | Success | 2 |
| 2011 | Austria | Kalkalpen | 1 | 0 | Success | 2 |
| 2011 | Austria | Kalkalpen | 0 | 1 | Success | 2 |
| 2013 | Austria | Kalkalpen | 1 | 0 | Success | 2 |
| 2014 | SE-Alps | Tarvisio | 1 | 1 | Success | 2 |
| 2017 | Austria | Kalkalpen | 1 | 1 | Success | 2 |
| 2019 | Dinaric | Risnjak | 0 | 1 | Success | 2* |
| 2019 | Dinaric | Loski potok | 0 | 1 | Success | 2* |
| 2020 | Dinaric | Paklenica | 0 | 1 | Success | 2* |
| 2020 | Dinaric | Snežnik | 0 | 1 | Success | 2* |
| 2020 | Dinaric | Loski potok | 0 | 1 | Success | 2* |
| 2020 | Dinaric | Velebit | 0 | 1 | Success | 2* |
| 2020 | Dinaric | Sneznik | 0 | 1 | Success | 2* |
| 2021 | SE-Alps | Triglav | 2 | 1 | Success | 2* |
| 2021 | SE-Alps | Nomenj-Gorjuše | 1 | 1 | Success | 2* |
| 2021 | Dinaric | Velebit | 0 | 1 | Success | 2* |
| 2022 | Dinaric | Velebit | 0 | 1 | Success | 2* |
| 2022 | Dinaric | Snežnik | 0 | 1 | Success | 2* |
| 2022 | Austria | Kalkalpen | 0 | 1 | Success | 2 |
| 2023 | Dinaric | Plitvice | 0 | 1 | Success | 2* |
| 2023 | Dinaric | Nomenj-Gorjuše | 0 | 1 | Success | 2* |
| 2023 | SE-Alps | Tarvisio | 3 | 2 | Success | 2* |
| 2023 | Dinaric | Snežnik | 1 | 0 | Success | 2* |

**References**

Alpine Convention. 2015. Available from https://www.atlas.alpconv.org/layers/geonode_data:geonode:RSA_V_AK_LAU2_2013_Inds_1_7_Population_densit (accessed October 3, 2025).

Bauduin S et al. 2021. Eurasian lynx populations in Western Europe: What prospects for the next 50 years?DOI: 10.1101/2021.10.22.465393. Available from http://biorxiv.org/lookup/doi/10.1101/2021.10.22.465393 (accessed October 11, 2023).

Becker T. 2013. Modeling Eurasian lynx (Lynx lynx) distribution and estimation of patch and population size in the Alps. Unviersity of London.

Belotti E et al. 2023. Lynx monitoring report for the Boheman-Bavarian-Austrian lynx population in 2019/2020. Page 36.

Breitenmoser U. 1998. Large predators in the Alps: The fall and rise of man’s competitors. Biological Conservation **83**:279–289.

Breitenmoser U, Breitenmoser-Würsten C. 2008. Der Luchs: Ein grossraubtier in der kulturlandschaft.

Breitenmoser U, Breitenmoser-Würsten C, Capt S, Molinari-Jobin A, Molinari P, Zimmermann F. 2007. Conservation of the lynx Lynx lynx in the Swiss Jura Mountains. Wildlife Biology **13**:340–355.

Breitenmoser U, Breitenmoser-Würsten C, Okarma H, Kaphegyi T, Kaphegyi-Wallmann U, Müller UM. 2000. Action plan for the conservation of the Eurasian Lynx in Europe (Lynx Lynx). Strasbourg.

Breitenmoser U, Haller H. 1993. Patterns of Predation by Reintroduced European Lynx in the Swiss Alps. The Journal of Wildlife Management **57**:135.

Breitenmoser U, Kavczensky P, Dötterer M, Breitenmoser‐Würsten C, Capt S, Bernhart F, Liberek M. 1993. Spatial organization and recruitment of lynx (*Lynx lynx*) in a re‐introduced population in the Swiss Jura Mountains. Journal of Zoology **231**:449–464.

Breitenmoser-Würsten C, Vandel J-M, Zimmermann F, Breitenmoser U. 2007. Demography of lynx Lynx lynx in the Jura Mountains. Wildlife Biology **13**:381–392.

Breitenmoser-Würsten C, Zimmermann F, Ryser A, Capt S, Laass J, Seigenthaler A, Breitenmoser U. 2001. Untersuchungen zur Luchspopulation in den Nordwestalpen der Schweiz 1997–2000, KORA Bericht 9. Page 88. 9. Stiftung KORA.

Bundesamt für Landestopographie. (n.d.). Alpen. Available from https://www.eda.admin.ch/aboutswitzerland/de/home/umwelt/geografie/alpen.html.

Bundesamt für Umwelt BAFU. (n.d.). Waldfläche in der Schweiz. Available from https://www.bafu.admin.ch/bafu/de/home/themen/wald/fachinformationen/waldzustand-und-waldfunktionen/waldflaeche-in-der-schweiz.html.

Capt S. 2007. Monitoring and Distribution of the Lynx Lynx Lynx in the Swiss Jura Mountains. Wildlife Biology **13**:356–364.

Colucci RR. 2016. Geomorphic influence on small glacier response to post-Little Ice Age climate warming: Julian Alps, Europe. Earth Surf. Process. Landforms **41**:1227–1240.

Čop J, Frković A. 1998. The re-introduction of the lynx in Slovenia and its present status in Slovenia and Croatia. Hystrix the Italian Journal of Mammalogy **10**:65–76.

Dellagiacoma F et al. 2016. The Statement On the Value of Alpine Forests and the Alpine Convention’s Protocol on Mountain Forests in the framework of the international forestry policies beyond 2015. Permanent Secretariat of the Alpine Convention. Available from http://www.alpconv.org/en/organization/groups/WGForest/Documents/Alpine_Forest_2015.pdf.

European Topic Centre on Land Cover & Environment Satellite Data Centre. 2018. Corine Land Cover 2018 (CLC). Kiruna, Sweden. Available from https://doi.org/10.2909/960998c1-1870-4e82-8051-6485205ebbac.

Ferreras P, Aldama JJ, Beltrán JF, Delibes M. 1992. Rates and causes of mortality in a fragmented population of Iberian lynx Felis pardina Temminck, 1824. Biological Conservation **61**:197–202.

Fležar U et al. 2023. Surveillance of the reinforcement process of the Dinaric - SE Alpine lynx population in the lynx-monitoring year 2021-2022. Page 73. Technical report. Ljubljana.

Fležar U, Pičulin A, Bartol M, Černe R, Stergar M, Krofel M, Potočnik H, Kljun F. 2019. Eurasian lynx (*Lynx Lynx*) monitoring with camera traps in Slovenia in 2018-2019. Pages 1–16.

Fuxjäger C. 2014. Der Luchs im Kalkalpen 2013. Pages 1–10. Kalkalpen Nationalpark.

Fuxjäger C. 2020. Der Luchs im Kalkalpen 2019. Kalkalpen Nationalpark.

Fuxjäger DC. 2012. Der Luchs im Nationalpark Kalkalpen 2011. Pages 1–10. Kalkalpen Nationalpark, Kalkalpen.

Fuxjäger DC. 2013. Der Luchs im Nationalpark Kalkalpen 2012. Pages 1–11. Kalkalpen Nationalpark.

Fuxjäger DC. 2015. Der Luchs im Nationalpark Kalkalpen.

Fuxjäger DC. 2016. Der Luchs im Nationalpark Kalkalpen 2015. Pages 1–21. Kalkalpen Nationalpark.

Fuxjäger DC. 2021. Der Luchs im Nationalpark Kalkalpen 2020. Pages 1–13. Kalkalpen Nationalpark.

Gaillard J-M, Nilsen EB, Odden J, Andrén H, Linnell JDC. 2014. One size fits all: Eurasian lynx females share a common optimal litter size. Journal of Animal Ecology **83**:107–115.

Gaona P, Ferreras P, Delibes M. 1998. Dynamics and viability of a metapopulation of the endangered Ibreian lynx (*LYNX PARDINUS*). Ecological Monographs **68**:349–370.

Gimenez O, Gatti S, Duchamp C, Germain E, Laurent A, Zimmermann F, Marboutin E. 2019. Spatial density estimates of Eurasian lynx ( *Lynx lynx* ) in the French Jura and Vosges Mountains. Ecology and Evolution **9**:11707–11715.

Gomerčić T, Topličanec I, Slijepčević V, Blašković S, Selanec I, Budinski I, Tomaić J, Kusak J, Ivanov G, Sindičić M. 2021. Distribution and minimum population size of Eurasian lynx (Lynx lynx) in Croatia in the period 2018-2020. Šumarski list **145**:525–533.

Grimm V et al. 2006. A standard protocol for describing individual-based and agent-based models. Ecological Modelling **198**:115–126.

Grimm V, Berger U, DeAngelis DL, Polhill JG, Giske J, Railsback SF. 2010. The ODD protocol: A review and first update. Ecological Modelling **221**:2760–2768.

Güthlin D, Knauer F, Kneib T, Küchenhoff H, Kaczensky P, Rauer G, Jonozovič M, Mustoni A, Jerina K. 2011. Estimating habitat suitability and potential population size for brown bears in the Eastern Alps. Biological Conservation **144**:1733–1741.

Heurich M, Brand TTG, Kaandorp MY, Šustr P, Müller J, Reineking B. 2015. Country, Cover or Protection: What Shapes the Distribution of Red Deer and Roe Deer in the Bohemian Forest Ecosystem? PLOS ONE **10**:e0120960.

Heurich M, Schultze-Naumburg J, Piacenza N, Magg N, Červený J, Engleder T, Herdtfelder M, Sladova M, Kramer-Schadt S. 2018. Illegal hunting as a major driver of the source-sink dynamics of a reintroduced lynx population in Central Europe. Biological Conservation **224**:355–365.

Jedrzejewski W, Jedrzejewska B, Okarma H, Schmidt K, Bunevich AN, Milkowski L. 1996. Population dynamics (1869–1994), demography, and home ranges of the lynx in Bialowieza Primeval Forest (Poland and Belarus). Ecography **19**:122–138.

Jewgenow K, Painer J, Amelkina O, Dehnhard M, Goeritz F. 2014. Lynx reproduction – Long-lasting life cycle of corpora lutea in a feline species. Reproductive Biology **14**:83–88.

Jobin A, Molinari P, Breitenmoser U. 2000. Prey spectrum, prey preference and consumption rates of Eurasian lynx in the Swiss Jura Mountains. Acta Theriologica **45**:243–252.

Kaczensky P, Chapron G. 2012. Status, management and distribution of large carnivores – bear, lynx, wolf & wolverine – in Europe.

Kaczensky P, Linnell JDC, Djuro H, Von Arx M, Andren H, Breitenmoser U, Boitani L. 2021. Distribution of large carnivores in Europe 2012 - 2016: Distribution maps for Brown bear, Eurasian lynx, Grey wolf, and Wolverine. Dryad.

Klar N, Herrmann M, Kramer-Schadt S. 2006. Effects of roads on a founder population of lynx in the biosphere reserve „Pfälzerwald – Vosges du Nord”. Naturschutz und Landschaftsplanung.

Kramer-Schadt S, Revilla E, Wiegand T. 2005. Lynx reintroductions in fragmented landscapes of Germany: Projects with a future or misunderstood wildlife conservation? Biological Conservation **125**:169–182.

Kramer-Schadt S, Revilla E, Wiegand T, Breitenmoser U. 2004. Fragmented landscapes, road mortality and patch connectivity: modelling influences on the dispersal of Eurasian lynx: Lynx dispersal in fragmented landscapes. Journal of Applied Ecology **41**:711–723.

Kramer-Schadt S, S. Kaiser T, Frank K, Wiegand T. 2011. Analyzing the effect of stepping stones on target patch colonisation in structured landscapes for Eurasian lynx. Landscape Ecology **26**:501–513.

Krofel M, Huber D, Kos I. 2011. Diet of Eurasian lynx Lynx lynx in the northern Dinaric Mountains (Slovenia and Croatia): Importance of edible dormouse Glis glis as alternative prey. Acta Theriologica **56**:315–322.

Kubala J et al. 2024. Factors shaping home ranges of Eurasian lynx (Lynx lynx) in the Western Carpathians. Scientific Reports **14**:21600.

Kunz F, Grand LL, Tremblay-Otis F, Breitenmoser-Würsten C, Breitenmoser U, Zimmermann F. 2019. Fang-Wiederfang-Schätzung der Abundanz und Dichte des Luchses im Jura Nord Ib im Winter 2018/19 KORA Bericht 86. 86. Stiftung KORA.

Magg N, Müller J, Heibl C, Hackländer K, Wölfl S, Wölfl M, Bufka L, Červený J, Heurich M. 2015. Habitat availability is not limiting the distribution of the Bohemian–Bavarian lynx *Lynx lynx* population. Oryx **50**:742–752.

Magg N, Müller J, Heibl C, Hackländer K, Wölfl S, Wölfl M, Bufka L, Červený J, Heurich M. 2016. Habitat availability is not limiting the distribution of the Bohemian-Bavarian lynx Lynx lynx population. Oryx **50**:742–752.

Mináriková T et al. 2023. Lynx Monitoring Report for Bohemian-Bavarian-Austrian lynx population for Lynx year 2017. Pages 1–20. Report prepared within the 3Lynx Project.

Molinari-Jobin A, Zimmermann F, Angst Ch, Breitenmoser-Würsten C, Capt S, Breitenmoser U. 2006. Status and distribution of the lynx in the Swiss Alps 2000–2004. Acta Biological Slovenica **49**:3–11.

Molinari-Jobin A, Zimmermann F, Breitenmoser-Würsten C, Capt S, Breitenmoser U. 2001. Present status and distribution of the lynx in the Swiss Alps. Hystrix the Italian Journal of Mammalogy **12**:17–27.

Molinari-Jobin A, Zimmermann F, Ryser A, Breitenmoser-Würsten C, Capt S, Breitenmoser U, Molinari P, Haller H, Eyholzer R. 2007. Variation in diet, prey selectivity and home-range size of Eurasian lynx Lynx lynx in Switzerland. Wildlife Biology **13**:393–405.

Müller J, Wölfl M, Wölfl S, Müller DWH, Hothorn T, Heurich M. 2014. Protected areas shape the spatial distribution of a European lynx population more than 20 years after reintroduction. Biological Conservation **177**:210–217.

Nagel TA, Mikac S, Dolinar M, Klopcic M, Keren S, Svoboda M, Diaci J, Boncina A, Paulic V. 2017. The natural disturbance regime in forests of the Dinaric Mountains: A synthesis of evidence. Forest Ecology and Management **388**:29–42.

Open Street Map. 2020. Geofabrik OSM dataset. Europe. Available from https://download.geofabrik.de/.

Pe’er G, Kramer-Schadt S. 2008. Incorporating the perceptual range of animals into connectivity models. Ecological Modelling **213**:73–85.

Potočnik H, Skrbinšek T, Kos I. 2009. The reintroduced Dinaric lynx population dynamics in PVA simulation: The 30 years retrospection and the future viability. Acta Biologica Slovenica **52**:3–18.

Premier J et al. 2025. Survival of Eurasian lynx in the human‐dominated landscape of Europe. Conservation Biology:e14439.

QGIS Development Team. 2020. QGIS Geographic Insformation System. Available from https://qgis.org/en/site/index.html.

R Core Team. 2023. R: A Language and Environment for Statistical Computing. R Foundation for Statistical Computing, Vienna, Austria.

Schadt S et al. 2002a. Assessing the suitability of central European landscapes for the reintroduction of Eurasian lynx: Lynx habitat suitability. Journal of Applied Ecology **39**:189–203.

Schadt S, Knauer F, Kaczensky P, Revilla E, Wiegand T, Trepl L. 2002b. Rule-based assessment of suitable habtitat and patch connectivity for the Eurasian lynx. Ecological Applications **12**:1469–1483.

Sindičić M, Sinanović N, Majić Skrbinšek A, Huber D, Kunovac S, Kos I. 2009. Legal status and management of the dinaric lynx popualation status. Veterinaria **58**:229–238.

Stanisa C, Koren I, Adamic iha. 2001. Situation and distribution of the lynx (Lynx lynx L.) in Slovenia from 1995-1999. Hystrix the Italian Journal of Mammalogy **12**:43–51.

Stiftung KORA. 2022, June 14. Bestand. Available from https://www.kora.ch/de/arten/luchs/bestand.

UNEP-WCMC and IUCN. 2020. The World Database on Protected Areas (WDPA). Cambridge, UK. Available from www.protectedplanet.net.

Vandel J-M, Stahl P. 2005. Distribution trend of the Eurasian lynx *Lynx lynx* populations in France. mamm **69**:145–158.

Vogt K, Korner‐Nievergelt F, Signer S, Zimmermann F, Marti I, Ryser A, Molinari‐Jobin A, Breitenmoser U, Breitenmoser‐Würsten Ch. 2025. Long‐Term Changes in Survival of Eurasian Lynx in Three Reintroduced Populations in Switzerland. Ecology and Evolution **15**:e71095.

Von Arx M, Breitenmoser-Würsten C, Zimmermann F, Breitenmoser U. 2004. Status and conservation of the Eurasian lynx (Lynx lynx) in Europe in 2001, KORA Bericht No. 19. 19. Stiftung KORA.

Von Arx M, Kaczensky P, Linnell JDC, Lars T, Breitenmoser-Würsten C, Boitani L, Breitenmoser U. 2021. Conservation status of the Eurasian lynx in West and Central Europe. CATnews Special Issue **14**:5–8.

Wölfl M, Červený J, Koubek P, Heurich M, Habel H, Huber T, Poost W. 2001. Distribution and status of lynx in the border region between Czech Republic, Germany and Austria. Acta Theriologica **46**:181–194.

Wölfl S et al. 2015a. Status and distribution of the transboundary lynx population of Czech Republic, Bavaria and Austria in the lynx year 2013. Page 21. Project Report of the Trans Lynx Project.

Wölfl S et al. 2015b. Status and dsitribution of the transboundary lynx popualtion of Czech Republic, Bavaria and Austria in the lynx year 2014. Page 12. Project Report of the Trans Lynx Project.

Wölfl S et al. 2023. Lynx monitoring report for the Boheminan-Bavarian-Austria lynx population in 2018/2019. Updated version of the report released in the year 2020. Page 29. 3Lynx project.

Zimmermann F, Breitenmoser U. 2007. Potential distribution and population size of the Eurasian lynx Lynx lynx in the jura Mountains and possible corridors to adjacent ranges. Wildlife Biology **13**:406–416.

Zimmermann F, Breitenmoser-Würsten C, Breitenmoser U. 2007. Importance of dispersal for the expansion of a Eurasian lynx *Lynx lynx* population in a fragmented landscape. Oryx **41**:358–368.

Zimmermann F, Meylan L, Frey O, Breitenmoser-Wursten C, Breitenmoser U, Kunz F. 2018. Abondance et densité du lynx dans le Sud du Jura suisse : estimation par capture-recapture photographique dans le sous-compartiment Ia, durant l’hiver 2017/18 KORA Rapport 80. Page 23. 80. Stiftung KORA.

Zupan Hajna N. 2019. Dinaric karst—Geography and geology. Pages 353–362 Encyclopedia of Caves. Elsevier. Available from https://linkinghub.elsevier.com/retrieve/pii/B978012814124300039X (accessed September 12, 2023).

**Appendix 2. Results**

**Model calibration**

**Appendix S9** Population sizes of resident (nRes) and dispersing (nDisp) lynx of field reports and model simulations by year and lynx population for each simulation phase. Population sizes of simulated data are reported as mean and standard deviation (Mean (SD)) and the median. For transboundary lynx populations, the international identification code of the country (ISO) where the population monitoring took place is indicated when the monitoring did not cover the full range of the population.

|  |  |  | Report | | | Simulated | | | | | |  |
| --- | --- | --- | --- | --- | --- | --- | --- | --- | --- | --- | --- | --- |
|  |  |  |  | | | **Res** | | **Disp** | | **Total** | |  |
| **Phase** | **Population** | **Year** | **Res** | **Disp** | **Pop. Size [CI]** | **Mean (SD)** | **Median** | **Mean (SD)** | **Median** | **Mean (SD)** | **Median** | **Reference** |
| 2 | Austria^a^ | 2011 |  |  | 1-4 | 1 (1) | 1 | 1 (1) | 1 | 2 (1) | 2 | Fuxjäger (2012) |
| 2 | Austria^a^ | 2012 | 3 |  | 1-4 | 1 (1) | 1 | 1 (1) | 1 | 2 (1) | 2 | Fuxjäger, (2013) |
| 2 | Austria^a^ | 2013 | 3 | 2 | 5 | 2 (1) | 2 | 1 (1) | 1 | 3 (2) | 2 | Fuxjäger (2014) |
| 2 | Austria^a^ | 2014 | 4 | 1 | 5 | 2 (1) | 2 | 1 (1) | 1 | 3 (2) | 2 | Fuxjäger, (2015) |
| 2 | Austria^a^ | 2015 | 2 | 3 | 5 | 2 (2) | 1 | 1 (1) | 1 | 3 (2) | 2 | Fuxjäger (2016) |
| 2 | Austria^a^ | 2019 |  |  | 6 | 3 (3) | 2 | 1 (1) | 1 | 4 (3) | 3 | Fuxjäger (2020) |
| 2 | Austria^a^ | 2020 |  |  | 6 | 3 (3) | 2 | 2 (2) | 2 | 5 (4) | 4 | Fuxjäger (2021) |
| 1 | BBA | 1990 | 18 |  |  | 22 (9) | 22 | 5 (3) | 4 | 27 (10) | 27 | Wölfl et al. (2001) |
| 1 | BBA | 1991 | 24 |  |  | 28 (12) | 27 | 6 (4) | 6 | 34 (14) | 33 | Wölfl et al. (2001) |
| 1 | BBA | 1992 | 32 |  |  | 35 (15) | 34 | 7 (5) | 7 | 43 (18) | 42 | Wölfl et al. (2001) |
| 1 | BBA | 1993 | 41 |  |  | 43 (18) | 42 | 10 (7) | 9 | 53 (23) | 50 | Wölfl et al. (2001) |
| 1 | BBA | 1994 | 51 |  |  | 51 (13) | 52 | 13 (10) | 11 | 65 (27) | 62 | Wölfl et al. (2001) |
| 2 | BBA | 1995 | 61 |  |  | 63 (6) | 63 | 10 (5) | 10 | 73 (8) | 73 | Wölfl et al. (2001) |
| 2 | BBA | 1996 | 69 |  |  | 71 (7) | 72 | 16 (6) | 16 | 87 (9) | 88 | Wölfl et al. (2001) |
| 2 | BBA | 1997 | 66 |  |  | 63 (7) | 64 | 21 (6) | 21 | 84 (10) | 84 | Wölfl et al. (2001) |
| 2 | BBA | 1998 | 68 |  |  | 66 (7) | 66 | 21 (7) | 21 | 87 (10) | 86 | Wölfl et al. (2001) |
| 2 | BBA | 2013 |  |  | 63^b^ [63-86] | 85 (14) | 85 | 23 (7) | 24 | 108 (18) | 108 | Wölfl et al., (2015) |
| 2 | BBA | 2014 |  |  | 59^c^ [59-83] | 87 (15) | 88 | 24 (7) | 23 | 111 (20) | 113 | Wölfl et al., (2015b) |
| 2 | BBA | 2017 |  |  | 110 | 92 (15) | 93 | 24 (8) | 24 | 116 (20) | 118 | Mináriková et al., (2023) |
| 2 | BBA | 2018 |  |  | 121 | 92 (16) | 94 | 26 (9) | 25 | 118 (23) | 119 | Wölfl et al., (2023) |
| 2 | BBA | 2019 |  |  | 133 | 94 (17) | 96 | 27 (10) | 26 | 121 (24) | 122 | Belotti et al., (2023) |
| 2 | Dinaric | 1995 |  |  | ~95 - ~140 | 102 (9) | 104 | 12 (5) | 12 | 114 (11) | 115 | Čop & Frković (1998);  Potočnik et al. (2009) |
| 2 | Dinaric (SI) | 1999 |  |  | 40 (10) | 94 (13) | 94 | 19 (7) | 18 | 113 (15) | 112 | Stanisa, et al. (2001) |
| 2 | Dinaric | 2008 |  |  | 130 | 95 (17) | 94 | 16 (6) | 17 | 112 (19) | 110 | Sindičić et al. (2009) |
| 2 | Dinaric (SI) | 2019 | ≥ 19 |  |  | 101 (23) | 102 | 16 (7) | 16 | 118 (27) | 116 | Fležar et al. (2019) |
| 2 | Dinaric (HR) | 2020 |  |  | 89-108 | 103 (24) | 103 | 19 (7) | 18 | 121 (28) | 120 | Gomerčić et al. (2021) |
| 2 | Jura Mts. | 1998 |  |  | 51-70 | 63 (10) | 63 | 11 (4) | 12 | 74 (12) | 74 | Zimmermann & Breitenmoser (2007) |
| 2 | Jura Mts. | 2001 |  |  | 56-78 | 74 (11) | 74 | 17 (6) | 16 | 91 (14) | 91 | Capt, (2007); Von Arx et al., (2004) |
| 2 | Jura Mts. (FR^d^) | 2002 | 60 |  | 115 | 77 (11) | 77 | 19 (7) | 18 | 95 (16) | 95 | Vandel & Stahl (2005) |
| 2 | Jura Mts. (FR^d^) | 2016 |  |  | 92 | 98 (10) | 97 | 36 (9) | 36 | 135 (15) | 134 | Gimenez et al. (2019) |
| 2 | Jura Mts. | 2016 |  |  | 140 | 98 (10) | 97 | 36 (9) | 36 | 135 (15) | 134 | Von Arx et al. (2021) |
| 2 | Jura Mts. | 2018 |  |  | 130-143 | 100 (9) | 100 | 37 (9) | 37 | 137 (14) | 138 | Kunz et al., (2019);  Zimmermann et al., (2018) |
| 1 | NW Alps | 1990 | 35-40 |  |  | 36 (25) | 34 | 11 (8) | 8 | 46 (32) | 42 | Haller et al 1992 as cited in Breitenmoser (1998) |
| 2 | NW Alps | 1995 | < 50 |  |  | 40 (28) | 40 | 11 (9) | 10 | 51 (36) | 52 | Breitenmoser (1998) |
| 2 | NW Alps | 2000 |  |  | 55-59 | 48 (9) | 48 | 12 (5) | 11 | 60 (11) | 60 | Breitenmoser-Würsten et al., (2001) |
| 2 | NW Alps | 2001 |  |  | ~70 | 57 (12) | 58 | 16 (6) | 16 | 73 (15) | 73 | Molinari-Jobin et al., (2001) |
| 2 | NW Alps | 2004 |  |  | 60-90 | 66 (15) | 65 | 19 (6) | 18 | 85 (19) | 85 | Molinari-Jobin et al., (2006) |
| 2 | NW Alps | 2010 |  |  | 96-104 | 90 (19) | 92 | 30 (9) | 28 | 120 (25) | 122 | Kaczensky & Chapron (2012) |
| 2 | NW Alps | 2016 |  |  | 163 | 112 (22) | 111 | 39 (13) | 38 | 151 (31) | 152 | Von Arx et al. (2021) |
| 2 | NW Alps | 2019 |  |  | 179 (8) | 124 (22) | 123 | 46 (14) | 44 | 170 (32) | 169 | Stiftung KORA, (2022) |
| 2 | SE Alps | 1999 |  |  | ~10 | 9 (5) | 9 | 2 (2) | 2 | 11 (5) | 11 | Stanisa, et al. (2001) |

^a^ Area of the Kalkalpen National Park

^b^ Minimum population size in a monitored area of 7.700 km^2^

^c^ Minimum population size in a monitored area of 13.000 km^2^

^d^ Lynx in the French Jura Mts. represent 2/3 of the total Jura Mts. Population.

**Population distribution**

All simulated lynx populations basically matched their respective reported distributions of phases 1 and 2 except the Dinaric. The simulated Dinaric lynx population did not fully cover the entire distribution range but matched the overall distribution patterns (Appendix S10). All simulated populations matched the reported population sizes (Appendix S9).


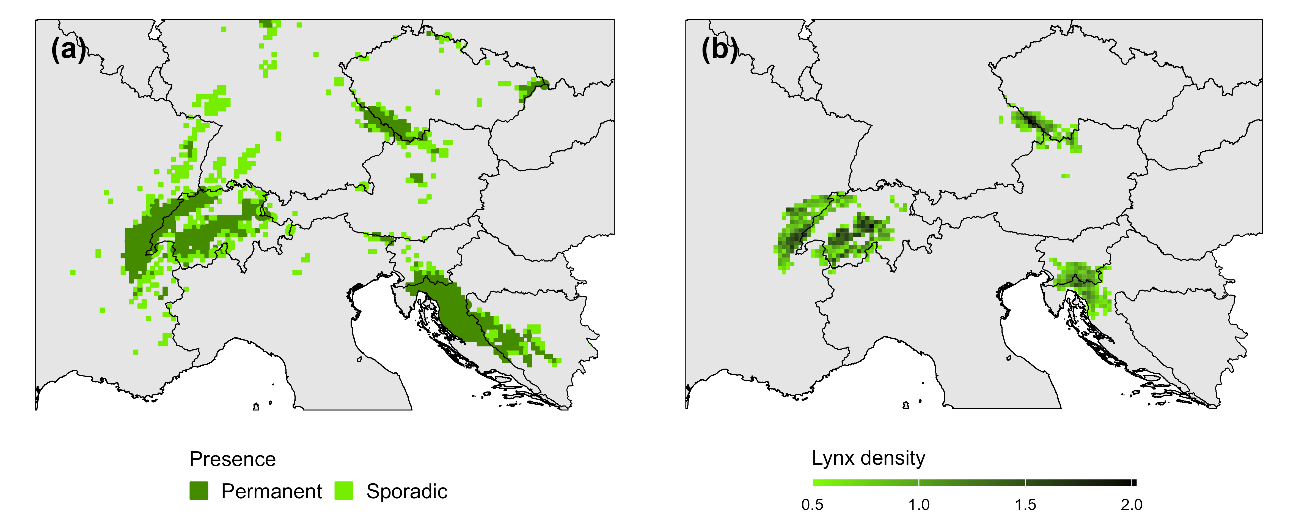


**Appendix S10** Real and simulated lynx population distributions in a 10x10 km grid. (a) shows the reported distributions of lynx populations in 2012-2016 (modified after Kaczensky et al. 2021). (b) shows the simulated lynx population density and distribution in 2016 (green to black palette).

**Simulated mortality**

**Appendix S11** Survival rates per population of dispersing and resident lynx resulting from our model simulations (Simulated survival rate (SD)) for the pre-release (1995–2018) and post-release (2019-2040) periods of the simulation in scenario 2. For the pre-release period we include survival estimates retrieved from field studies, indicated by the superindex.

| **Years** | **Population** | **Status** | **Simulated Survival rate (SD)** | **Estimated Survival from publications** |
| --- | --- | --- | --- | --- |
| 1995-2018 | Austria | Dispersers | 0.65 (0.057) | Not assessed |
|  |  | Residents | 0.815 (0.206) | Not assessed |
|  | BBA | Dispersers | 0.491 (0.046) | Not assessed |
|  |  | Residents | 0.707 (0.053) | 0.86^a^; 0.69-0.7^b^ |
|  | Dinaric | Dispersers | 0.456 (0.028) | 0.82^a^; <0.62^c^ |
|  |  | Residents | 0.652 (0.044) | 0.95^a^; <0.83^c^ |
|  | Jura Mts. | Dispersers | 0.51 (0.02) | 0.65^a^, 0.53^d^; ~0.75^e^ |
|  |  | Residents | 0.73 (0.025) | 0.84^a^ ; 0.76^e^ |
|  | NW Alps | Dispersers | 0.555 (0.024) | 0.76^a^;~0.83^e^ |
|  |  | Residents | 0.737 (0.035) | 0.75^a^; 0.8 ^e^ |
|  | SE Alps | Dispersers | 0.485 (0.035) | 0.82^a^; <0.62^c^ |
|  |  | Residents | 0.656 (0.046) | 0.95^a^; <0.83^c^ |
| 2019-2040 | Austria | Dispersers | 0.592 (0.024) |  |
|  |  | Residents | 0.744 (0.014) |  |
|  | BBA | Dispersers | 0.488 (0.007) |  |
|  |  | Residents | 0.691 (0.004) |  |
|  | Dinaric | Dispersers | 0.445 (0.009) |  |
|  |  | Residents | 0.642 (0.004) |  |
|  | Jura Mts. | Dispersers | 0.528 (0.007) |  |
|  |  | Residents | 0.721 (0.004) |  |
|  | NW Alps | Dispersers | 0.569 (0.007) |  |
|  |  | Residents | 0.724 (0.003) |  |
|  | SE Alps | Dispersers | 0.461 (0.015) |  |
|  |  | Residents | 0.658 (0.007) |  |

^a^ Premier et al. (2025), results are based on telemetry data and thus survival estimates do not account for the effects of low genetic diversity.

^b^ Heurich et al. (2018), survival rate combined resident and disperser mortality derived from a modelling approach.

c Potočnik et al. (2009); survival rate used to recreate population development using a demographic model

^c^ Breitenmoser-Würsten et al. (2007), survival rates estimated from telemetry data

^d^ Vogt et al. (2025), survival rate estimated after combining data from carcasses, captured individuals, telemetry data, and systematic and opportunistic camera-trap monitoring.

**Patch colonization**

**Appendix S12** Coefficient values of the linear mixed effects model analyzing the effect of immigration on the local population size, based on immigration load (NewImm), patch size (Area), year (Year) and population size of residents (meanRes).

| Effect | Group | Term | Estimate (SE) | df | statistic | *p* |
| --- | --- | --- | --- | --- | --- | --- |
| Fixed |  | NewImm | 49.2446 (4.5864) | 953 | 10.7370 | <0.001*** |
|  |  | NewImm^2^ | 9.2111 (1.904) | 953 | 4.8377 | <0.001*** |
|  |  | Area | 0.001 (1e-04) | 22 | 8.5431 | <0.001*** |
|  |  | Year | 8e-04 (0.0011) | 953 | 0.7406 | 0.4591. |
|  |  | meanRes | 0.9009 (0.0063) | 953 | 142.6294 | <0.001*** |
|  |  | NewImm : Area | -0.0076 (0.0012) | 953 | -6.4070 | <0.001*** |
|  |  | NewImm^2^ : Area | -0.0015 (4e-04) | 953 | -3.6056 | <0.001*** |
| Random | LastPatchNo^b^ | sdIntercept | 0.8304 |  |  |  |
|  | Residual | sdObservation | 0.3303 |  |  |  |

^a^ *p* < 0.05(*); *p* < 0.01 (**); *p* < 0.001(***).

^b^ Represents „patch ID“ and was included as a random intercept.

**References**

Alpine Convention. 2015. Available from https://www.atlas.alpconv.org/layers/geonode_data:geonode:RSA_V_AK_LAU2_2013_Inds_1_7_Population_densit (accessed October 3, 2025).

Bauduin S et al. 2021. Eurasian lynx populations in Western Europe: What prospects for the next 50 years?DOI: 10.1101/2021.10.22.465393. Available from http://biorxiv.org/lookup/doi/10.1101/2021.10.22.465393 (accessed October 11, 2023).

Becker T. 2013. Modeling Eurasian lynx (Lynx lynx) distribution and estimation of patch and population size in the Alps. Unviersity of London.

Belotti E et al. 2023. Lynx monitoring report for the Boheman-Bavarian-Austrian lynx population in 2019/2020. Page 36.

Breitenmoser U. 1998. Large predators in the Alps: The fall and rise of man’s competitors. Biological Conservation **83**:279–289.

Breitenmoser U, Breitenmoser-Würsten C. 2008. Der Luchs: Ein grossraubtier in der kulturlandschaft.

Breitenmoser U, Breitenmoser-Würsten C, Capt S, Molinari-Jobin A, Molinari P, Zimmermann F. 2007. Conservation of the lynx Lynx lynx in the Swiss Jura Mountains. Wildlife Biology **13**:340–355.

Breitenmoser U, Breitenmoser-Würsten C, Okarma H, Kaphegyi T, Kaphegyi-Wallmann U, Müller UM. 2000. Action plan for the conservation of the Eurasian Lynx in Europe (Lynx Lynx). Strasbourg.

Breitenmoser U, Haller H. 1993. Patterns of Predation by Reintroduced European Lynx in the Swiss Alps. The Journal of Wildlife Management **57**:135.

Breitenmoser U, Kavczensky P, Dötterer M, Breitenmoser‐Würsten C, Capt S, Bernhart F, Liberek M. 1993. Spatial organization and recruitment of lynx (*Lynx lynx*) in a re‐introduced population in the Swiss Jura Mountains. Journal of Zoology **231**:449–464.

Breitenmoser-Würsten C, Vandel J-M, Zimmermann F, Breitenmoser U. 2007. Demography of lynx Lynx lynx in the Jura Mountains. Wildlife Biology **13**:381–392.

Breitenmoser-Würsten C, Zimmermann F, Ryser A, Capt S, Laass J, Seigenthaler A, Breitenmoser U. 2001. Untersuchungen zur Luchspopulation in den Nordwestalpen der Schweiz 1997–2000, KORA Bericht 9. Page 88. 9. Stiftung KORA.

Bundesamt für Landestopographie. (n.d.). Alpen. Available from https://www.eda.admin.ch/aboutswitzerland/de/home/umwelt/geografie/alpen.html.

Bundesamt für Umwelt BAFU. (n.d.). Waldfläche in der Schweiz. Available from https://www.bafu.admin.ch/bafu/de/home/themen/wald/fachinformationen/waldzustand-und-waldfunktionen/waldflaeche-in-der-schweiz.html.

Capt S. 2007. Monitoring and Distribution of the Lynx Lynx Lynx in the Swiss Jura Mountains. Wildlife Biology **13**:356–364.

Colucci RR. 2016. Geomorphic influence on small glacier response to post-Little Ice Age climate warming: Julian Alps, Europe. Earth Surf. Process. Landforms **41**:1227–1240.

Čop J, Frković A. 1998. The re-introduction of the lynx in Slovenia and its present status in Slovenia and Croatia. Hystrix the Italian Journal of Mammalogy **10**:65–76.

Dellagiacoma F et al. 2016. The Statement On the Value of Alpine Forests and the Alpine Convention’s Protocol on Mountain Forests in the framework of the international forestry policies beyond 2015. Permanent Secretariat of the Alpine Convention. Available from http://www.alpconv.org/en/organization/groups/WGForest/Documents/Alpine_Forest_2015.pdf.

European Topic Centre on Land Cover & Environment Satellite Data Centre. 2018. Corine Land Cover 2018 (CLC). Kiruna, Sweden. Available from https://doi.org/10.2909/960998c1-1870-4e82-8051-6485205ebbac.

Ferreras P, Aldama JJ, Beltrán JF, Delibes M. 1992. Rates and causes of mortality in a fragmented population of Iberian lynx Felis pardina Temminck, 1824. Biological Conservation **61**:197–202.

Fležar U et al. 2023. Surveillance of the reinforcement process of the Dinaric - SE Alpine lynx population in the lynx-monitoring year 2021-2022. Page 73. Technical report. Ljubljana.

Fležar U, Pičulin A, Bartol M, Černe R, Stergar M, Krofel M, Potočnik H, Kljun F. 2019. Eurasian lynx (*Lynx Lynx*) monitoring with camera traps in Slovenia in 2018-2019. Pages 1–16.

Fuxjäger C. 2014. Der Luchs im Kalkalpen 2013. Pages 1–10. Kalkalpen Nationalpark.

Fuxjäger C. 2020. Der Luchs im Kalkalpen 2019. Kalkalpen Nationalpark.

Fuxjäger DC. 2012. Der Luchs im Nationalpark Kalkalpen 2011. Pages 1–10. Kalkalpen Nationalpark, Kalkalpen.

Fuxjäger DC. 2013. Der Luchs im Nationalpark Kalkalpen 2012. Pages 1–11. Kalkalpen Nationalpark.

Fuxjäger DC. 2015. Der Luchs im Nationalpark Kalkalpen.

Fuxjäger DC. 2016. Der Luchs im Nationalpark Kalkalpen 2015. Pages 1–21. Kalkalpen Nationalpark.

Fuxjäger DC. 2021. Der Luchs im Nationalpark Kalkalpen 2020. Pages 1–13. Kalkalpen Nationalpark.

Gaillard J-M, Nilsen EB, Odden J, Andrén H, Linnell JDC. 2014. One size fits all: Eurasian lynx females share a common optimal litter size. Journal of Animal Ecology **83**:107–115.

Gaona P, Ferreras P, Delibes M. 1998. Dynamics and viability of a metapopulation of the endangered Ibreian lynx (*LYNX PARDINUS*). Ecological Monographs **68**:349–370.

Gimenez O, Gatti S, Duchamp C, Germain E, Laurent A, Zimmermann F, Marboutin E. 2019. Spatial density estimates of Eurasian lynx ( *Lynx lynx* ) in the French Jura and Vosges Mountains. Ecology and Evolution **9**:11707–11715.

Gomerčić T, Topličanec I, Slijepčević V, Blašković S, Selanec I, Budinski I, Tomaić J, Kusak J, Ivanov G, Sindičić M. 2021. Distribution and minimum population size of Eurasian lynx (Lynx lynx) in Croatia in the period 2018-2020. Šumarski list **145**:525–533.

Grimm V et al. 2006. A standard protocol for describing individual-based and agent-based models. Ecological Modelling **198**:115–126.

Grimm V, Berger U, DeAngelis DL, Polhill JG, Giske J, Railsback SF. 2010. The ODD protocol: A review and first update. Ecological Modelling **221**:2760–2768.

Güthlin D, Knauer F, Kneib T, Küchenhoff H, Kaczensky P, Rauer G, Jonozovič M, Mustoni A, Jerina K. 2011. Estimating habitat suitability and potential population size for brown bears in the Eastern Alps. Biological Conservation **144**:1733–1741.

Heurich M, Brand TTG, Kaandorp MY, Šustr P, Müller J, Reineking B. 2015. Country, Cover or Protection: What Shapes the Distribution of Red Deer and Roe Deer in the Bohemian Forest Ecosystem? PLOS ONE **10**:e0120960.

Heurich M, Schultze-Naumburg J, Piacenza N, Magg N, Červený J, Engleder T, Herdtfelder M, Sladova M, Kramer-Schadt S. 2018. Illegal hunting as a major driver of the source-sink dynamics of a reintroduced lynx population in Central Europe. Biological Conservation **224**:355–365.

Jedrzejewski W, Jedrzejewska B, Okarma H, Schmidt K, Bunevich AN, Milkowski L. 1996. Population dynamics (1869–1994), demography, and home ranges of the lynx in Bialowieza Primeval Forest (Poland and Belarus). Ecography **19**:122–138.

Jewgenow K, Painer J, Amelkina O, Dehnhard M, Goeritz F. 2014. Lynx reproduction – Long-lasting life cycle of corpora lutea in a feline species. Reproductive Biology **14**:83–88.

Jobin A, Molinari P, Breitenmoser U. 2000. Prey spectrum, prey preference and consumption rates of Eurasian lynx in the Swiss Jura Mountains. Acta Theriologica **45**:243–252.

Kaczensky P, Chapron G. 2012. Status, management and distribution of large carnivores – bear, lynx, wolf & wolverine – in Europe.

Kaczensky P, Linnell JDC, Djuro H, Von Arx M, Andren H, Breitenmoser U, Boitani L. 2021. Distribution of large carnivores in Europe 2012 - 2016: Distribution maps for Brown bear, Eurasian lynx, Grey wolf, and Wolverine. Dryad.

Klar N, Herrmann M, Kramer-Schadt S. 2006. Effects of roads on a founder population of lynx in the biosphere reserve „Pfälzerwald – Vosges du Nord”. Naturschutz und Landschaftsplanung.

Kramer-Schadt S, Revilla E, Wiegand T. 2005. Lynx reintroductions in fragmented landscapes of Germany: Projects with a future or misunderstood wildlife conservation? Biological Conservation **125**:169–182.

Kramer-Schadt S, Revilla E, Wiegand T, Breitenmoser U. 2004. Fragmented landscapes, road mortality and patch connectivity: modelling influences on the dispersal of Eurasian lynx: Lynx dispersal in fragmented landscapes. Journal of Applied Ecology **41**:711–723.

Kramer-Schadt S, S. Kaiser T, Frank K, Wiegand T. 2011. Analyzing the effect of stepping stones on target patch colonisation in structured landscapes for Eurasian lynx. Landscape Ecology **26**:501–513.

Krofel M, Huber D, Kos I. 2011. Diet of Eurasian lynx Lynx lynx in the northern Dinaric Mountains (Slovenia and Croatia): Importance of edible dormouse Glis glis as alternative prey. Acta Theriologica **56**:315–322.

Kubala J et al. 2024. Factors shaping home ranges of Eurasian lynx (Lynx lynx) in the Western Carpathians. Scientific Reports **14**:21600.

Kunz F, Grand LL, Tremblay-Otis F, Breitenmoser-Würsten C, Breitenmoser U, Zimmermann F. 2019. Fang-Wiederfang-Schätzung der Abundanz und Dichte des Luchses im Jura Nord Ib im Winter 2018/19 KORA Bericht 86. 86. Stiftung KORA.

Magg N, Müller J, Heibl C, Hackländer K, Wölfl S, Wölfl M, Bufka L, Červený J, Heurich M. 2015. Habitat availability is not limiting the distribution of the Bohemian–Bavarian lynx *Lynx lynx* population. Oryx **50**:742–752.

Magg N, Müller J, Heibl C, Hackländer K, Wölfl S, Wölfl M, Bufka L, Červený J, Heurich M. 2016. Habitat availability is not limiting the distribution of the Bohemian-Bavarian lynx Lynx lynx population. Oryx **50**:742–752.

Mináriková T et al. 2023. Lynx Monitoring Report for Bohemian-Bavarian-Austrian lynx population for Lynx year 2017. Pages 1–20. Report prepared within the 3Lynx Project.

Molinari-Jobin A, Zimmermann F, Angst Ch, Breitenmoser-Würsten C, Capt S, Breitenmoser U. 2006. Status and distribution of the lynx in the Swiss Alps 2000–2004. Acta Biological Slovenica **49**:3–11.

Molinari-Jobin A, Zimmermann F, Breitenmoser-Würsten C, Capt S, Breitenmoser U. 2001. Present status and distribution of the lynx in the Swiss Alps. Hystrix the Italian Journal of Mammalogy **12**:17–27.

Molinari-Jobin A, Zimmermann F, Ryser A, Breitenmoser-Würsten C, Capt S, Breitenmoser U, Molinari P, Haller H, Eyholzer R. 2007. Variation in diet, prey selectivity and home-range size of Eurasian lynx Lynx lynx in Switzerland. Wildlife Biology **13**:393–405.

Müller J, Wölfl M, Wölfl S, Müller DWH, Hothorn T, Heurich M. 2014. Protected areas shape the spatial distribution of a European lynx population more than 20 years after reintroduction. Biological Conservation **177**:210–217.

Nagel TA, Mikac S, Dolinar M, Klopcic M, Keren S, Svoboda M, Diaci J, Boncina A, Paulic V. 2017. The natural disturbance regime in forests of the Dinaric Mountains: A synthesis of evidence. Forest Ecology and Management **388**:29–42.

Open Street Map. 2020. Geofabrik OSM dataset. Europe. Available from https://download.geofabrik.de/.

Pe’er G, Kramer-Schadt S. 2008. Incorporating the perceptual range of animals into connectivity models. Ecological Modelling **213**:73–85.

Potočnik H, Skrbinšek T, Kos I. 2009. The reintroduced Dinaric lynx population dynamics in PVA simulation: The 30 years retrospection and the future viability. Acta Biologica Slovenica **52**:3–18.

Premier J et al. 2025. Survival of Eurasian lynx in the human‐dominated landscape of Europe. Conservation Biology:e14439.

QGIS Development Team. 2020. QGIS Geographic Insformation System. Available from https://qgis.org/en/site/index.html.

R Core Team. 2023. R: A Language and Environment for Statistical Computing. R Foundation for Statistical Computing, Vienna, Austria.

Schadt S et al. 2002a. Assessing the suitability of central European landscapes for the reintroduction of Eurasian lynx: Lynx habitat suitability. Journal of Applied Ecology **39**:189–203.

Schadt S, Knauer F, Kaczensky P, Revilla E, Wiegand T, Trepl L. 2002b. Rule-based assessment of suitable habtitat and patch connectivity for the Eurasian lynx. Ecological Applications **12**:1469–1483.

Sindičić M, Sinanović N, Majić Skrbinšek A, Huber D, Kunovac S, Kos I. 2009. Legal status and management of the dinaric lynx popualation status. Veterinaria **58**:229–238.

Stanisa C, Koren I, Adamic iha. 2001. Situation and distribution of the lynx (Lynx lynx L.) in Slovenia from 1995-1999. Hystrix the Italian Journal of Mammalogy **12**:43–51.

Stiftung KORA. 2022, June 14. Bestand. Available from https://www.kora.ch/de/arten/luchs/bestand.

UNEP-WCMC and IUCN. 2020. The World Database on Protected Areas (WDPA). Cambridge, UK. Available from www.protectedplanet.net.

Vandel J-M, Stahl P. 2005. Distribution trend of the Eurasian lynx *Lynx lynx* populations in France. mamm **69**:145–158.

Vogt K, Korner‐Nievergelt F, Signer S, Zimmermann F, Marti I, Ryser A, Molinari‐Jobin A, Breitenmoser U, Breitenmoser‐Würsten Ch. 2025. Long‐Term Changes in Survival of Eurasian Lynx in Three Reintroduced Populations in Switzerland. Ecology and Evolution **15**:e71095.

Von Arx M, Breitenmoser-Würsten C, Zimmermann F, Breitenmoser U. 2004. Status and conservation of the Eurasian lynx (Lynx lynx) in Europe in 2001, KORA Bericht No. 19. 19. Stiftung KORA.

Von Arx M, Kaczensky P, Linnell JDC, Lars T, Breitenmoser-Würsten C, Boitani L, Breitenmoser U. 2021. Conservation status of the Eurasian lynx in West and Central Europe. CATnews Special Issue **14**:5–8.

Wölfl M, Červený J, Koubek P, Heurich M, Habel H, Huber T, Poost W. 2001. Distribution and status of lynx in the border region between Czech Republic, Germany and Austria. Acta Theriologica **46**:181–194.

Wölfl S et al. 2015a. Status and distribution of the transboundary lynx population of Czech Republic, Bavaria and Austria in the lynx year 2013. Page 21. Project Report of the Trans Lynx Project.

Wölfl S et al. 2015b. Status and dsitribution of the transboundary lynx popualtion of Czech Republic, Bavaria and Austria in the lynx year 2014. Page 12. Project Report of the Trans Lynx Project.

Wölfl S et al. 2023. Lynx monitoring report for the Boheminan-Bavarian-Austria lynx population in 2018/2019. Updated version of the report released in the year 2020. Page 29. 3Lynx project.

Zimmermann F, Breitenmoser U. 2007. Potential distribution and population size of the Eurasian lynx Lynx lynx in the jura Mountains and possible corridors to adjacent ranges. Wildlife Biology **13**:406–416.

Zimmermann F, Breitenmoser-Würsten C, Breitenmoser U. 2007. Importance of dispersal for the expansion of a Eurasian lynx *Lynx lynx* population in a fragmented landscape. Oryx **41**:358–368.

Zimmermann F, Meylan L, Frey O, Breitenmoser-Wursten C, Breitenmoser U, Kunz F. 2018. Abondance et densité du lynx dans le Sud du Jura suisse : estimation par capture-recapture photographique dans le sous-compartiment Ia, durant l’hiver 2017/18 KORA Rapport 80. Page 23. 80. Stiftung KORA.

Zupan Hajna N. 2019. Dinaric karst—Geography and geology. Pages 353–362 Encyclopedia of Caves. Elsevier. Available from https://linkinghub.elsevier.com/retrieve/pii/B978012814124300039X (accessed September 12, 2023).

**Appendix 3.**

We describe the model following the ODD protocol for individual-based models (Overview, Design Concepts, Detail; Grimm et al. 2006, 2010). The ODD protocol is based on the protocol from Kramer-Schadt et al. (2011) and has been adapted for our study.

**Purpose**

We used an individual-based, spatially-explicit population simulation model to assess the spread, colonization success, viability of six reintroduced lynx populations and population connectivity in heterogeneous landscapes under different simulation scenarios. The simulated lynx populations are those in the Bohemian-Bavarian-Austrian Forest, Dinaric Mountains, Jura Mountains, Austrian and NW Alps and SE Alps. The model has been published in two original papers (Kramer-Schadt et al. 2004, 2005) and has been used for a variety of purposes, e.g. for assessing the additional impact of roads (Klar et al. 2006) or perceptual range (Pe’er & Kramer-Schadt 2008) on population connectivity and viability. Here, we describe the common basic model; specific settings, simulation experiments and changes to the model rules are described in the respective publications.

**State variables and scales**

The model consists of two sub-models, a demographic model of lynx considering territory occupation, reproduction, and mortality, and a dispersal model that links the demographic processes. Both sub-models are landscape-specific. Demographic parameters stem from published data of long-term field studies in fragmented landscapes in Switzerland, Poland and Spain (Ferreras et al. 1992; Jedrzejewski et al. 1996; Breitenmoser-Würsten et al. 2001). The reproductive rate parameter was obtained from multiple lynx populations and studies (Bauduin et al. 2021). The original dispersal module (Kramer-Schadt et al. 2004) was calibrated with field data from dispersing lynx collected in the Swiss Jura Mountains (Breitenmoser et al. 1993).

The basic entity of the model is the individual. The state variables of the lynx individuals are (1) sex, (2) age, (3) location (x-y coordinates), and (4) demographic status (disperser or resident). The model is based on raster (gridded) maps over which demographic and dispersal processes take place. Each cell of the map has a resolution of 1km2 (spatial resolution) which corresponds with the animal’s perceptual range as the smallest spatial unit. The extent of the study area is 1382x931 cells. The habitat map is the basis for the spatial processes of dispersal and territory search. It is further complimented with a linear barrier map and added mortality map. These maps together constitute a landscape of habitat suitability and mortality risk (see Appendix 1, “Maps of the model: overview” for details). Cells represent functional landscape types for lynx and are classed in (a) suitable for breeding, (b) suitable for dispersal, (c) avoided, but used occasionally, and (d) barriers (Schadt et al. 2002b). Time steps represent one day to capture the variability in daily dispersal distance in concert with the landscape variability. Starting simulation time (in years) varies between populations according to the first year of lynx reintroductions in the respective populations. Ending simulation time is 20 years into the future.

The model operates at two temporal resolutions. The dispersal module for dispersers operates at a one-day resolution (daily temporal resolution). Dispersers move every day through the landscape according to a set of behavioral rules culminating in variability in movement. Thus, they have an every-day mortality probability that accumulates at the end of the year. Contrarily, the demographic sub-model for residents occurs at one-felid year timespan, defined by the lynx’ reproduction, from the birth of the kittens until they disperse and search for a territory (Zimmermann et al., 2005) (annual temporal resolution). Processes of survival and reproduction probabilities for residents are determined with annual probabilities (yearly temporal resolution).

**Process overview and scheduling**

At the beginning of each model time step (year), the number of resident and non-resident males and females on the landscape map are determined. All non-residents older than 1 year disperse and search for territories (see Dispersal Sub-model). Each day, non-resident individuals disperse a certain number of steps with the direction depending on the underlying landscape type, search for a territory and undergo a daily mortality probability. The spatially explicit processes of dispersal and territory selection in the model depend upon local habitat quality within the immediate surrounding of the animals’ location. If dispersing individuals survive (probabilistic event depending on daily dispersal mortality M_disp_), they settle or continue dispersing in the following year. At the end of each daily time step, the location of the dispersing individuals is updated. If the disperser has found and occupied a territory, the status is set to resident. Next, the demographic processes of the residents come into play (see Demographic sub-model). At the end of each year, the occupied territories of the residents and the age and status of each lynx are updated (Appendix S13).

**Design concepts**

The model considers lynx demography, dispersal, territory selection and occupation and interaction of the landscape types with these ecological processes. The behavior and demography of the lynx are imposed by reaction towards the landscape types as well as by status-dependent parameters. Stochasticity is included to represent demographic and environmental noise. Allee effects are considered in the demographic sub-model: only when male and female lynx have overlapping territories is reproduction considered.

Emergence.

Mortality, reproduction, dispersal and territory establishment are a result of an interaction between the individual movement and the habitat and stochasticity. Therefore, territory occupation, population density, population sizes and connectivity are all processes emerging from the decision-making process of the individual as it moves through the landscape.

Dispersing individuals move a maximum number of steps (grids in the habitat raster layer) per day according to a set of movement parameters, and their movement is defined by a set of rules (Kramer-Schadt et al. 2004, 2005). Therefore, dispersal distances emerge from the decision-making process of the individual during their movement. The decision-making process will also affect, for example, the time until individuals find enough suitable habitat to establish a territory, the number of linear barriers they will cross during their movement and consequently the survival probability and connectivity between populations. Territory establishment differs between females and males. Territory establishment in females depends on availability of breeding habitat in the landscape, while males search for female territories that are not overlapping with other males’ territories. Therefore, mortality and survival rates, reproduction, connectivity and population distribution emerges in the model from the interaction between the habitat and decision-making process of the individual.

Observation.

The model records individual-level processes that build into population-level processes throughout the entire simulation. For each timestep and repetition (iteration of a timestep), the model records for every individual the intrinsic and spatial characteristics. Intrinsic characteristics include the individual ID, sex, age, life stage (or status, resident or disperser), ID of the mother and the father, reproduction, home range size, mortality (dead or alive) and cause of mortality (baseline, traffic, added mortality). Spatial characteristics of the individual include the initial (at the beginning of the timestep) and last (at the end location in the landscape (xy cells) and the patch ID associated to these locations. Variable like home range size are life-stage specific and will only be recorded for residents, and remain empty for dispersing individuals. Likewise, resident individuals do not move through the landscape, they remain in the same xy cell and ‘occupy’ several territory cells until they lose the territory, and thus the start and end locations in each timestep are the same for residents.

By tracking the fate and spatial location of each individual (each with its own intrinsic characteristics) throughout the simulation demographic parameters, like population size and extinction rate in a designated patch and connectivity between patches, can be derived from the model.

**Initialization**

The 50 years since the first reintroductions were divided into two phases reflecting the trend of the successfully reintroduced populations. The first phase simulates the settlement of lynx and population expansion. The second phase simulates the stagnation and projection into the future). Different demographic parameters were used in the two phases. In phase 1, initial population size is given by the number of reintroduced individuals (Breitenmoser & Breitenmoser-Würsten 2008, unpublished data). All individuals start as dispersers. In phase 2, the initial population size is a continuation of phase 1, thus the best snapshot of the population in phase 1.

**Input**

The model does not include any external model or data files of driving environmental variables.

**Sub-models**

*Demographic sub-model.* This sub-model controls the individuals with resident status. Territory occupation, reproduction, and resident mortality are processes happening on an annual level. Each resident female whose territory is overlapped by that of a male reproduces with a certain probability (Pbirth; Appendix S14). We set the probability of having one or two cubs surviving their first year to 0.5 and the sex ratio to 1:1 Annual resident mortality (Mres) is also a probabilistic event. The cells belonging to a resident’s territory are kept if the individual survives. The probability of resident lynx abandoning their territory while they are alive is very low because lynx have high fidelity to their home range (Kubala et al. 2024).

*Dispersal sub-model (including territory searching behavior).* Each day, each dispersing lynx is assigned a certain number of movement steps *s* based on a probability *P*(*s*) using a power function with an exponent *x* and parameter smax that determines the maximum number of steps that a dispersing lynx can cover during a single day: *P(s)* = (1 − ((*s* − 1)/(smax − 1)))*^x^*. Parameter s is based on the model calibration with field data (Appendix S14). The spatial unit of dispersal is one movement step, i.e. 1 km^2^ grid cell. In each step, individuals survey their eight-cell neighborhood and make decisions based on this information (see below). Their choice of direction is comprised of two components: the probability of leaving preferred dispersal habitat by stepping into the matrix (Pmatrix, Appendix S14), and a correlation factor determining the probability of continuing with the same direction as their previous movement within a day (*P_C_*, Appendix S14). The hierarchy is a preference of dispersal habitat over a persistent movement forward, with the first direction of every day chosen randomly. Within a day, the next cell is chosen based on the preference for dispersal habitat and the avoidance of matrix. If the neighborhood of a dispersing lynx, comprising the origin cell and its 8 neighbors, contains only matrix or dispersal habitat cells, the probability of choosing one of these cells is random (i.e., 1/9). However, if the neighborhood is a mixture of dispersal habitat and matrix, we consider the preference for dispersal habitat as follows: The number of matrix cells nmat_mat_ within the neighborhood is counted. The probability of leaving dispersal habitat Pleave is then dependent on the number of matrix cells around the origin cell multiplied by a factor Pmatrix (ranging from total avoidance of matrix [Pmatrix = 0] to randomly choosing any surrounding cell [Pmatrix = 1/9], with Pleave = nmat * Pmatrix. If an animal has stepped into the matrix, it is assigned a ‘memory’ of its last location in a dispersal habitat, toward which it returns should it fail to find a dispersal habitat cell within Pmaxmatrix = 10 steps. A daily mortality probability is included (Mdisp_,_ Appendix S14). We note that the mortality probability Mdisp is landscape-independent due to the absence of sufficient field-data regarding mortality risks in different landscape types.

We upscale the landscape in terms of territory searching behavior, i.e., each dispersing female needs to collect a certain amount of contagious cells of non-occupied breeding habitat (N_HRCells_), whereas males search for cells that are already occupied by females and can overlap up to 3 females. To include stochasticity in territory size we draw for each female a random number of cells from a uniform distribution N_HRCells_ between 42 and 105 cells. The simulated female then has to use this amount of cells as her territory. Once occupied, territory cells cannot be used by other females. In unoccupied areas the female that comes first has the best chance of occupying a territory. An overview of the model processes and schedule is found on Appendix S13.

**
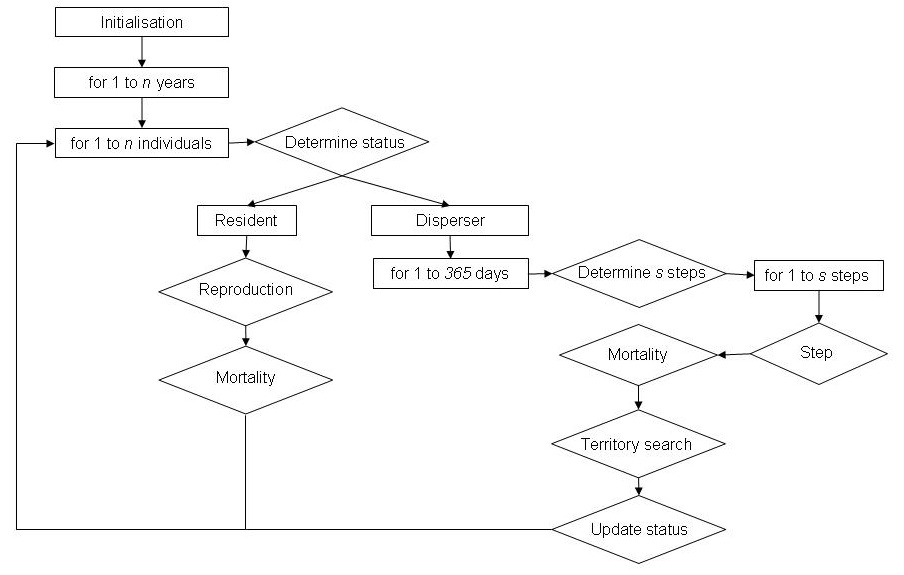
Appendix S13** Flowchart of the model. Rectangles indicate states and diamonds indicate process

**Appendix S14** Basic parameter values for the demographic and the dispersal sub-models. Values can change depending on the scenarios assessed in the respective publications. Asterisk indicates the parameters was calculated from field data.

| Sub-model | Symbol | Parameter value or range |
| --- | --- | --- |
| *Demographic sub-model* |  |  |
| Reproduction rate (=prob. of giving birth) | Pbirth* | 0.83 |
| Annual mortality probability of residents | Mres^a^ | 0.1 (~13% annual mortality rate) |
| *Dispersal sub-model* |  |  |
| Correlation factor | P_C_ ^c^ | 0.5 |
| Probability of stepping into matrix | Pmatrix ^c^ | 0.03 |
| Maximum number of steps an individual stays in matrix before returning | Pmaxmatrix^c^ | 10 |
| Maximum number of steps per day | smax_max_ ^c^ | 45 |
| Exponent of step distribution | x ^c^ | 11 |
| Daily mortality probability of dispersers | Mdisp^b,e^ | 0.0006 (~46% annual mortality rate) |

^a^ Ferreras et al. (1992)

^b^ Breitenmoser-Würsten et al. (2001)

^c^ Kramer-Schadt et al. (2004)

^e^Gaona et al. (1998)

**References**

Alpine Convention. 2015. Available from https://www.atlas.alpconv.org/layers/geonode_data:geonode:RSA_V_AK_LAU2_2013_Inds_1_7_Population_densit (accessed October 3, 2025).

Bauduin S et al. 2021. Eurasian lynx populations in Western Europe: What prospects for the next 50 years?DOI: 10.1101/2021.10.22.465393. Available from http://biorxiv.org/lookup/doi/10.1101/2021.10.22.465393 (accessed October 11, 2023).

Becker T. 2013. Modeling Eurasian lynx (Lynx lynx) distribution and estimation of patch and population size in the Alps. Unviersity of London.

Belotti E et al. 2023. Lynx monitoring report for the Boheman-Bavarian-Austrian lynx population in 2019/2020. Page 36.

Breitenmoser U. 1998. Large predators in the Alps: The fall and rise of man’s competitors. Biological Conservation **83**:279–289.

Breitenmoser U, Breitenmoser-Würsten C. 2008. Der Luchs: Ein grossraubtier in der kulturlandschaft.

Breitenmoser U, Breitenmoser-Würsten C, Capt S, Molinari-Jobin A, Molinari P, Zimmermann F. 2007. Conservation of the lynx Lynx lynx in the Swiss Jura Mountains. Wildlife Biology **13**:340–355.

Breitenmoser U, Breitenmoser-Würsten C, Okarma H, Kaphegyi T, Kaphegyi-Wallmann U, Müller UM. 2000. Action plan for the conservation of the Eurasian Lynx in Europe (Lynx Lynx). Strasbourg.

Breitenmoser U, Haller H. 1993. Patterns of Predation by Reintroduced European Lynx in the Swiss Alps. The Journal of Wildlife Management **57**:135.

Breitenmoser U, Kavczensky P, Dötterer M, Breitenmoser‐Würsten C, Capt S, Bernhart F, Liberek M. 1993. Spatial organization and recruitment of lynx (*Lynx lynx*) in a re‐introduced population in the Swiss Jura Mountains. Journal of Zoology **231**:449–464.

Breitenmoser-Würsten C, Vandel J-M, Zimmermann F, Breitenmoser U. 2007. Demography of lynx Lynx lynx in the Jura Mountains. Wildlife Biology **13**:381–392.

Breitenmoser-Würsten C, Zimmermann F, Ryser A, Capt S, Laass J, Seigenthaler A, Breitenmoser U. 2001. Untersuchungen zur Luchspopulation in den Nordwestalpen der Schweiz 1997–2000, KORA Bericht 9. Page 88. 9. Stiftung KORA.

Bundesamt für Landestopographie. (n.d.). Alpen. Available from https://www.eda.admin.ch/aboutswitzerland/de/home/umwelt/geografie/alpen.html.

Bundesamt für Umwelt BAFU. (n.d.). Waldfläche in der Schweiz. Available from https://www.bafu.admin.ch/bafu/de/home/themen/wald/fachinformationen/waldzustand-und-waldfunktionen/waldflaeche-in-der-schweiz.html.

Capt S. 2007. Monitoring and Distribution of the Lynx Lynx Lynx in the Swiss Jura Mountains. Wildlife Biology **13**:356–364.

Colucci RR. 2016. Geomorphic influence on small glacier response to post-Little Ice Age climate warming: Julian Alps, Europe. Earth Surf. Process. Landforms **41**:1227–1240.

Čop J, Frković A. 1998. The re-introduction of the lynx in Slovenia and its present status in Slovenia and Croatia. Hystrix the Italian Journal of Mammalogy **10**:65–76.

Dellagiacoma F et al. 2016. The Statement On the Value of Alpine Forests and the Alpine Convention’s Protocol on Mountain Forests in the framework of the international forestry policies beyond 2015. Permanent Secretariat of the Alpine Convention. Available from http://www.alpconv.org/en/organization/groups/WGForest/Documents/Alpine_Forest_2015.pdf.

European Topic Centre on Land Cover & Environment Satellite Data Centre. 2018. Corine Land Cover 2018 (CLC). Kiruna, Sweden. Available from https://doi.org/10.2909/960998c1-1870-4e82-8051-6485205ebbac.

Ferreras P, Aldama JJ, Beltrán JF, Delibes M. 1992. Rates and causes of mortality in a fragmented population of Iberian lynx Felis pardina Temminck, 1824. Biological Conservation **61**:197–202.

Fležar U et al. 2023. Surveillance of the reinforcement process of the Dinaric - SE Alpine lynx population in the lynx-monitoring year 2021-2022. Page 73. Technical report. Ljubljana.

Fležar U, Pičulin A, Bartol M, Černe R, Stergar M, Krofel M, Potočnik H, Kljun F. 2019. Eurasian lynx (*Lynx Lynx*) monitoring with camera traps in Slovenia in 2018-2019. Pages 1–16.

Fuxjäger C. 2014. Der Luchs im Kalkalpen 2013. Pages 1–10. Kalkalpen Nationalpark.

Fuxjäger C. 2020. Der Luchs im Kalkalpen 2019. Kalkalpen Nationalpark.

Fuxjäger DC. 2012. Der Luchs im Nationalpark Kalkalpen 2011. Pages 1–10. Kalkalpen Nationalpark, Kalkalpen.

Fuxjäger DC. 2013. Der Luchs im Nationalpark Kalkalpen 2012. Pages 1–11. Kalkalpen Nationalpark.

Fuxjäger DC. 2015. Der Luchs im Nationalpark Kalkalpen.

Fuxjäger DC. 2016. Der Luchs im Nationalpark Kalkalpen 2015. Pages 1–21. Kalkalpen Nationalpark.

Fuxjäger DC. 2021. Der Luchs im Nationalpark Kalkalpen 2020. Pages 1–13. Kalkalpen Nationalpark.

Gaillard J-M, Nilsen EB, Odden J, Andrén H, Linnell JDC. 2014. One size fits all: Eurasian lynx females share a common optimal litter size. Journal of Animal Ecology **83**:107–115.

Gaona P, Ferreras P, Delibes M. 1998. Dynamics and viability of a metapopulation of the endangered Ibreian lynx (*LYNX PARDINUS*). Ecological Monographs **68**:349–370.

Gimenez O, Gatti S, Duchamp C, Germain E, Laurent A, Zimmermann F, Marboutin E. 2019. Spatial density estimates of Eurasian lynx ( *Lynx lynx* ) in the French Jura and Vosges Mountains. Ecology and Evolution **9**:11707–11715.

Gomerčić T, Topličanec I, Slijepčević V, Blašković S, Selanec I, Budinski I, Tomaić J, Kusak J, Ivanov G, Sindičić M. 2021. Distribution and minimum population size of Eurasian lynx (Lynx lynx) in Croatia in the period 2018-2020. Šumarski list **145**:525–533.

Grimm V et al. 2006. A standard protocol for describing individual-based and agent-based models. Ecological Modelling **198**:115–126.

Grimm V, Berger U, DeAngelis DL, Polhill JG, Giske J, Railsback SF. 2010. The ODD protocol: A review and first update. Ecological Modelling **221**:2760–2768.

Güthlin D, Knauer F, Kneib T, Küchenhoff H, Kaczensky P, Rauer G, Jonozovič M, Mustoni A, Jerina K. 2011. Estimating habitat suitability and potential population size for brown bears in the Eastern Alps. Biological Conservation **144**:1733–1741.

Heurich M, Brand TTG, Kaandorp MY, Šustr P, Müller J, Reineking B. 2015. Country, Cover or Protection: What Shapes the Distribution of Red Deer and Roe Deer in the Bohemian Forest Ecosystem? PLOS ONE **10**:e0120960.

Heurich M, Schultze-Naumburg J, Piacenza N, Magg N, Červený J, Engleder T, Herdtfelder M, Sladova M, Kramer-Schadt S. 2018. Illegal hunting as a major driver of the source-sink dynamics of a reintroduced lynx population in Central Europe. Biological Conservation **224**:355–365.

Jedrzejewski W, Jedrzejewska B, Okarma H, Schmidt K, Bunevich AN, Milkowski L. 1996. Population dynamics (1869–1994), demography, and home ranges of the lynx in Bialowieza Primeval Forest (Poland and Belarus). Ecography **19**:122–138.

Jewgenow K, Painer J, Amelkina O, Dehnhard M, Goeritz F. 2014. Lynx reproduction – Long-lasting life cycle of corpora lutea in a feline species. Reproductive Biology **14**:83–88.

Jobin A, Molinari P, Breitenmoser U. 2000. Prey spectrum, prey preference and consumption rates of Eurasian lynx in the Swiss Jura Mountains. Acta Theriologica **45**:243–252.

Kaczensky P, Chapron G. 2012. Status, management and distribution of large carnivores – bear, lynx, wolf & wolverine – in Europe.

Kaczensky P, Linnell JDC, Djuro H, Von Arx M, Andren H, Breitenmoser U, Boitani L. 2021. Distribution of large carnivores in Europe 2012 - 2016: Distribution maps for Brown bear, Eurasian lynx, Grey wolf, and Wolverine. Dryad.

Klar N, Herrmann M, Kramer-Schadt S. 2006. Effects of roads on a founder population of lynx in the biosphere reserve „Pfälzerwald – Vosges du Nord”. Naturschutz und Landschaftsplanung.

Kramer-Schadt S, Revilla E, Wiegand T. 2005. Lynx reintroductions in fragmented landscapes of Germany: Projects with a future or misunderstood wildlife conservation? Biological Conservation **125**:169–182.

Kramer-Schadt S, Revilla E, Wiegand T, Breitenmoser U. 2004. Fragmented landscapes, road mortality and patch connectivity: modelling influences on the dispersal of Eurasian lynx: Lynx dispersal in fragmented landscapes. Journal of Applied Ecology **41**:711–723.

Kramer-Schadt S, S. Kaiser T, Frank K, Wiegand T. 2011. Analyzing the effect of stepping stones on target patch colonisation in structured landscapes for Eurasian lynx. Landscape Ecology **26**:501–513.

Krofel M, Huber D, Kos I. 2011. Diet of Eurasian lynx Lynx lynx in the northern Dinaric Mountains (Slovenia and Croatia): Importance of edible dormouse Glis glis as alternative prey. Acta Theriologica **56**:315–322.

Kubala J et al. 2024. Factors shaping home ranges of Eurasian lynx (Lynx lynx) in the Western Carpathians. Scientific Reports **14**:21600.

Kunz F, Grand LL, Tremblay-Otis F, Breitenmoser-Würsten C, Breitenmoser U, Zimmermann F. 2019. Fang-Wiederfang-Schätzung der Abundanz und Dichte des Luchses im Jura Nord Ib im Winter 2018/19 KORA Bericht 86. 86. Stiftung KORA.

Magg N, Müller J, Heibl C, Hackländer K, Wölfl S, Wölfl M, Bufka L, Červený J, Heurich M. 2015. Habitat availability is not limiting the distribution of the Bohemian–Bavarian lynx *Lynx lynx* population. Oryx **50**:742–752.

Magg N, Müller J, Heibl C, Hackländer K, Wölfl S, Wölfl M, Bufka L, Červený J, Heurich M. 2016. Habitat availability is not limiting the distribution of the Bohemian-Bavarian lynx Lynx lynx population. Oryx **50**:742–752.

Mináriková T et al. 2023. Lynx Monitoring Report for Bohemian-Bavarian-Austrian lynx population for Lynx year 2017. Pages 1–20. Report prepared within the 3Lynx Project.

Molinari-Jobin A, Zimmermann F, Angst Ch, Breitenmoser-Würsten C, Capt S, Breitenmoser U. 2006. Status and distribution of the lynx in the Swiss Alps 2000–2004. Acta Biological Slovenica **49**:3–11.

Molinari-Jobin A, Zimmermann F, Breitenmoser-Würsten C, Capt S, Breitenmoser U. 2001. Present status and distribution of the lynx in the Swiss Alps. Hystrix the Italian Journal of Mammalogy **12**:17–27.

Molinari-Jobin A, Zimmermann F, Ryser A, Breitenmoser-Würsten C, Capt S, Breitenmoser U, Molinari P, Haller H, Eyholzer R. 2007. Variation in diet, prey selectivity and home-range size of Eurasian lynx Lynx lynx in Switzerland. Wildlife Biology **13**:393–405.

Müller J, Wölfl M, Wölfl S, Müller DWH, Hothorn T, Heurich M. 2014. Protected areas shape the spatial distribution of a European lynx population more than 20 years after reintroduction. Biological Conservation **177**:210–217.

Nagel TA, Mikac S, Dolinar M, Klopcic M, Keren S, Svoboda M, Diaci J, Boncina A, Paulic V. 2017. The natural disturbance regime in forests of the Dinaric Mountains: A synthesis of evidence. Forest Ecology and Management **388**:29–42.

Open Street Map. 2020. Geofabrik OSM dataset. Europe. Available from https://download.geofabrik.de/.

Pe’er G, Kramer-Schadt S. 2008. Incorporating the perceptual range of animals into connectivity models. Ecological Modelling **213**:73–85.

Potočnik H, Skrbinšek T, Kos I. 2009. The reintroduced Dinaric lynx population dynamics in PVA simulation: The 30 years retrospection and the future viability. Acta Biologica Slovenica **52**:3–18.

Premier J et al. 2025. Survival of Eurasian lynx in the human‐dominated landscape of Europe. Conservation Biology:e14439.

QGIS Development Team. 2020. QGIS Geographic Insformation System. Available from https://qgis.org/en/site/index.html.

R Core Team. 2023. R: A Language and Environment for Statistical Computing. R Foundation for Statistical Computing, Vienna, Austria.

Schadt S et al. 2002a. Assessing the suitability of central European landscapes for the reintroduction of Eurasian lynx: Lynx habitat suitability. Journal of Applied Ecology **39**:189–203.

Schadt S, Knauer F, Kaczensky P, Revilla E, Wiegand T, Trepl L. 2002b. Rule-based assessment of suitable habtitat and patch connectivity for the Eurasian lynx. Ecological Applications **12**:1469–1483.

Sindičić M, Sinanović N, Majić Skrbinšek A, Huber D, Kunovac S, Kos I. 2009. Legal status and management of the dinaric lynx popualation status. Veterinaria **58**:229–238.

Stanisa C, Koren I, Adamic iha. 2001. Situation and distribution of the lynx (Lynx lynx L.) in Slovenia from 1995-1999. Hystrix the Italian Journal of Mammalogy **12**:43–51.

Stiftung KORA. 2022, June 14. Bestand. Available from https://www.kora.ch/de/arten/luchs/bestand.

UNEP-WCMC and IUCN. 2020. The World Database on Protected Areas (WDPA). Cambridge, UK. Available from www.protectedplanet.net.

Vandel J-M, Stahl P. 2005. Distribution trend of the Eurasian lynx *Lynx lynx* populations in France. mamm **69**:145–158.

Vogt K, Korner‐Nievergelt F, Signer S, Zimmermann F, Marti I, Ryser A, Molinari‐Jobin A, Breitenmoser U, Breitenmoser‐Würsten Ch. 2025. Long‐Term Changes in Survival of Eurasian Lynx in Three Reintroduced Populations in Switzerland. Ecology and Evolution **15**:e71095.

Von Arx M, Breitenmoser-Würsten C, Zimmermann F, Breitenmoser U. 2004. Status and conservation of the Eurasian lynx (Lynx lynx) in Europe in 2001, KORA Bericht No. 19. 19. Stiftung KORA.

Von Arx M, Kaczensky P, Linnell JDC, Lars T, Breitenmoser-Würsten C, Boitani L, Breitenmoser U. 2021. Conservation status of the Eurasian lynx in West and Central Europe. CATnews Special Issue **14**:5–8.

Wölfl M, Červený J, Koubek P, Heurich M, Habel H, Huber T, Poost W. 2001. Distribution and status of lynx in the border region between Czech Republic, Germany and Austria. Acta Theriologica **46**:181–194.

Wölfl S et al. 2015a. Status and distribution of the transboundary lynx population of Czech Republic, Bavaria and Austria in the lynx year 2013. Page 21. Project Report of the Trans Lynx Project.

Wölfl S et al. 2015b. Status and dsitribution of the transboundary lynx popualtion of Czech Republic, Bavaria and Austria in the lynx year 2014. Page 12. Project Report of the Trans Lynx Project.

Wölfl S et al. 2023. Lynx monitoring report for the Boheminan-Bavarian-Austria lynx population in 2018/2019. Updated version of the report released in the year 2020. Page 29. 3Lynx project.

Zimmermann F, Breitenmoser U. 2007. Potential distribution and population size of the Eurasian lynx Lynx lynx in the jura Mountains and possible corridors to adjacent ranges. Wildlife Biology **13**:406–416.

Zimmermann F, Breitenmoser-Würsten C, Breitenmoser U. 2007. Importance of dispersal for the expansion of a Eurasian lynx *Lynx lynx* population in a fragmented landscape. Oryx **41**:358–368.

Zimmermann F, Meylan L, Frey O, Breitenmoser-Wursten C, Breitenmoser U, Kunz F. 2018. Abondance et densité du lynx dans le Sud du Jura suisse : estimation par capture-recapture photographique dans le sous-compartiment Ia, durant l’hiver 2017/18 KORA Rapport 80. Page 23. 80. Stiftung KORA.

Zupan Hajna N. 2019. Dinaric karst—Geography and geology. Pages 353–362 Encyclopedia of Caves. Elsevier. Available from https://linkinghub.elsevier.com/retrieve/pii/B978012814124300039X (accessed September 12, 2023).
